# Intercellular Variability in Protein Levels from Stochastic Expression and Noisy Cell Cycle Processes

**DOI:** 10.1371/journal.pcbi.1004972

**Published:** 2016-08-18

**Authors:** Mohammad Soltani, Cesar A. Vargas-Garcia, Duarte Antunes, Abhyudai Singh

**Affiliations:** 1 Electrical and Computer Engineering Department, University of Delaware, Newark, Delaware, United States of America; 2 Mechanical Engineering Department, Eindhoven University of Technology, Eindhoven, Netherlands; 3 Biomedical Engineering Department, University of Delaware, Newark, Delaware, United States of America; 4 Mathematical Sciences Department, University of Delaware, Newark, Delaware, United States of America; 5 Center for Bioinformatics and Computational Biology, University of Delaware, Newark, Delaware, United States of America; UCSD, UNITED STATES

## Abstract

Inside individual cells, expression of genes is inherently stochastic and manifests as cell-to-cell variability or noise in protein copy numbers. Since proteins half-lives can be comparable to the cell-cycle length, randomness in cell-division times generates additional intercellular variability in protein levels. Moreover, as many mRNA/protein species are expressed at low-copy numbers, errors incurred in partitioning of molecules between two daughter cells are significant. We derive analytical formulas for the total noise in protein levels when the cell-cycle duration follows a general class of probability distributions. Using a novel hybrid approach the total noise is decomposed into components arising from i) stochastic expression; ii) partitioning errors at the time of cell division and iii) random cell-division events. These formulas reveal that random cell-division times not only generate additional extrinsic noise, but also critically affect the mean protein copy numbers and intrinsic noise components. Counter intuitively, in some parameter regimes, noise in protein levels can decrease as cell-division times become more stochastic. Computations are extended to consider genome duplication, where transcription rate is increased at a random point in the cell cycle. We systematically investigate how the timing of genome duplication influences different protein noise components. Intriguingly, results show that noise contribution from stochastic expression is minimized at an optimal genome-duplication time. Our theoretical results motivate new experimental methods for decomposing protein noise levels from synchronized and asynchronized single-cell expression data. Characterizing the contributions of individual noise mechanisms will lead to precise estimates of gene expression parameters and techniques for altering stochasticity to change phenotype of individual cells.

## Author Summary

Inside individual cells, gene products often occur at low molecular counts and are subject to considerable stochastic fluctuations (noise) in copy numbers over time. An important consequence of noisy expression is that the level of a protein can vary considerably even among genetically identical cells exposed to the same environment. Such non-genetic phenotypic heterogeneity is physiologically relevant and critically influences diverse cellular processes. In addition to noise sources inherent in gene product synthesis, recent experimental studies have uncovered additional noise mechanisms that critically effect expression. For example, the time within the cell cycle when a gene duplicates, and the time taken to complete cell cycle are governed by random processes. The key contribution of this work is development of novel mathematical results quantifying how cell cycle-related noise sources combine with stochastic expression to drive intercellular variability in protein molecular counts. Derived formulas lead to many counterintuitive results, such as increasing randomness in the timing of cell division can lower noise in the level of a protein. Finally, these results inform experimental strategies to systematically dissect the contributions of different noise sources in the expression of a gene of interest.

## Introduction

**Fig 1:**
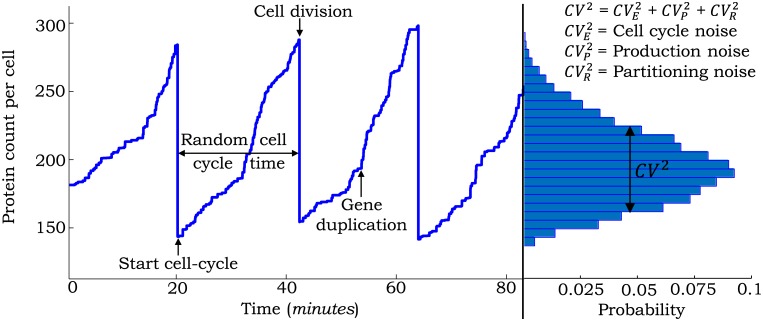
Sample trajectory of the protein level in a single cell with different sources of noise. Stochastically expressed proteins accumulate within the cell at a certain rate. At a random point in the cell cycle, gene duplication results in an increase in production rate. Stochastic cell-division events lead to random partitioning of protein molecules between two daughter cells with each cell receiving, on average, half the number of proteins in the mother cell just before division. The steady-state protein copy number distribution obtained from a large number of trajectories is shown on the right. The total noise in the protein level, as measured by the squared coefficient of variation (*CV*^2^) can be broken into contributions from individual noise mechanisms.

The level of a protein can deviate considerably from cell-to-cell, in spite of the fact that cells are genetically-identical and are in the same extracellular environment [1–3]. This intercellular variation or noise in protein counts has been implicated in diverse processes such as corrupting functioning of gene networks [4–6], driving probabilistic cell-fate decisions [7–12], buffering cell populations from hostile changes in the environment [13–16], and causing clonal cells to respond differently to the same stimulus [17–19]. An important source of noise driving random fluctuations in protein levels is stochastic gene expression due to the inherent probabilistic nature of biochemical processes [20–23]. Recent experimental studies have uncovered additional noise sources that affect protein copy numbers. For example, the time take to complete cell cycle (i.e., time between two successive cell-division events) has been observed to be stochastic across organisms [24–32]. Moreover, given that many proteins/mRNAs are present inside cells at low-copy numbers, errors incurred in partitioning of molecules between two daughter cells are significant [33–35]. Finally, the time at which a particular gene of interest is duplicated can also vary between cells [36, 37]. We investigate how such noise sources in the cell-cycle process combine with stochastic gene expression to generate intercellular variability in protein copy numbers (Fig 1).

Prior studies that quantify the effects of cell division on the protein noise level have been restricted to specific cases. For example, noise computations have been done in stochastic gene expression models, where cell divisions occur at deterministic time intervals [33, 38, 39]. Recently, we have analyzed a deterministic model of gene expression with random cell-division events [40]. Building up on this work, we formulate a mathematical model that couples stochastic expression of a stable protein with random cell-division events that follow a general class of probability distributions. Moreover, at the time of cell division, proteins are randomly partitioned between two daughter cells based on a framework that allows the partitioning errors to be higher or lower than as predicted by binomial partitioning. For this class of models, we derive an exact analytical formula for the protein noise level as quantified by the steady-state squared Coefficient of Variation (*CV*^2^). This formula is further decomposed into individual components representing contributions from different noise sources. A systematic investigation of this formula leads to novel insights, such as identification of regimes where increasing randomness in the timing of cell-division events decreases the protein noise level.

Next, we extend the above model to include genome-duplication events that increase the gene’s transcription rate [36, 41]. To our knowledge, this is the first study integrating randomness in the genome-duplication process with stochastic gene expression. An exact formula for the protein noise level is derived for this extended model and used to investigate how the timing of duplication affects different noise components. Counter intuitively, results show that doubling of the transcription rate within the cell cycle can lead to smaller fluctuations in protein levels as compared to a constant transcription rate through out the cell cycle. Finally, we discuss how formulas obtained in this study can be used to infer parameters and characterize the gene expression process from single-cell studies.

## Methods

### Coupling gene expression to cell division

We consider the standard model of stochastic gene expression [42, 43], where mRNAs are transcribed at exponentially distributed time intervals from a constitutive gene with rate *k*_*x*_. For the time being, we exclude genome duplication and the transcription rate is fixed throughout the cell cycle. Assuming short-lived mRNAs, each transcription event results in a burst of proteins [43–45]. The corresponding jump in protein levels is shown as

$$\begin{array}{c} x(t)\mapsto x(t)+B, \end{array}$$ (1)

where *x*(*t*) is the protein population count in the mother cell at time *t*, *B* is a random burst size drawn from a positively-valued distribution and represents the number of protein molecules synthesized in a single-mRNA lifetime. Motivated by observations in *E. coli* and mammalian cells, where many proteins have half-lives considerably longer than the cell-doubling time, we assume a stable protein with no active degradation [46–48]. Thus, proteins accumulate within the cell till the time of cell division, at which point they are randomly partitioned between two daughter cells.

Let cell division events occur at times *t*_*s*_, *s* ∈ {1, 2, …}. The cell-cycle time

$$T≔t_{s}-t_{s-1},$$ (2)

follows an arbitrary positively-valued probability distribution with the following mean and squared coefficient of variation (*CV*^2^)

$$\langle T\rangle =\langle t_{s}-t_{s-1}\rangle ,\;CV_{T}^{2}=\frac{\langle T^{2}\rangle -{\langle T\rangle }^{2}}{{\langle T\rangle }^{2}},$$ (3)

where 〈.〉 denotes expected value through out this paper. The random change in *x*(*t*) during cell division is given by

$$x\left(t_{s}\right)\mapsto x_{+}\left(t_{s}\right),$$ (4)

where *x*(*t*_*s*_) denotes the protein levels in the mother cell just before division and *x*_+_(*t*_*s*_) denotes the protein levels in one of the daughter cells just after division. Conditioned on *x*(*t*_*s*_), *x*_+_(*t*_*s*_) is assumed to have the following statistics

$$\langle x_{+}\left(t_{s}\right)|x\left(t_{s}\right)\rangle =\frac{x(t_{s})}{2},\;\langle x_{+}^{2}\left(t_{s}\right)-{\langle x_{+}\left(t_{s}\right)\rangle }^{2}|x\left(t_{s}\right)\rangle =\frac{\alpha x(t_{s})}{4}.$$ (5)

The first equation implies symmetric partitioning, i.e., on average each of the daughter cells inherits half the number protein molecules just before division. The second equation in Eq (5) describes the variance of *x*_+_(*t*_*s*_) and quantifies the error in partitioning of molecules through the non-negative parameter *α*. For example, *α* = 0 represents deterministic partitioning where *x*_+_(*t*_*s*_) = *x*(*t*_*s*_)/2 with probability equal to one. A more realistic model for partitioning is each molecule having an equal probability of being in the each daughter cell [49–51]. This results in a binomial distribution for *x*_+_(*t*_*s*_)

$$\text{Probability}\left\{x_{+}\left(t_{s}\right)=j|x\left(t_{s}\right)\right\}=\frac{x(t_{s})!}{j!(x\left(t_{s}\right)-j)!}{\left(\frac{1}{2}\right)}^{x(t_{s})},\;\;j\in \left\{0,1,\ldots ,x\left(t_{s}\right)\right\},$$ (6)

and corresponds to *α* = 1 in Eq (5). Interestingly, recent studies have shown that partitioning of proteins that form clusters or multimers can result in *α* > 1 in Eq (5), i.e., partitioning errors are much higher than as predicted by the binomial distribution [33, 39]. In contrast, if molecules push each other to opposite poles of the cell, then the partitioning errors will be smaller than as predicted by Eq (6) and *α* < 1.

**Fig 2:**
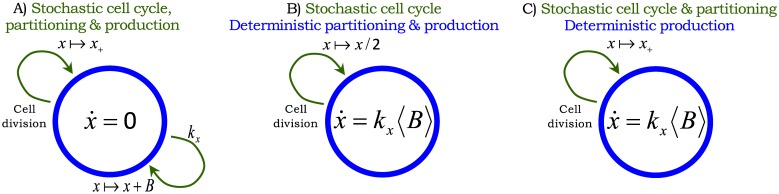
Stochastic models of gene expression with cell division. Arrows denote stochastic events that change the protein level by discrete jumps as shown in Eqs (1) and (4). The differential equation within the circle represents the time evolution of *x*(*t*) in between events. **A)** Model with all the different sources of noise: proteins are expressed in stochastic bursts, cell division occurs at random times, and molecules are partitioned between the two daughter cells based on Eq (5). The trivial dynamics $\dot{x}=0$ signifies that the protein level is constant in-between stochastic events. **B)** Hybrid model where randomness in cell-division events is the only source of noise. Protein production is modeled deterministic through a differential equation and partitioning errors are absent, i.e., *α* = 0 in Eq (5). **C)** Hybrid model where noise comes from both cell-division events and partitioning errors. Protein production is considered to be deterministic as in Fig 2B. Since *x*(*t*) is continuous here, *x*_+_(*t*_*s*_) has a positively-valued continuous distribution with same mean and variance as in Eq (5)

The model with all the different noise mechanisms (stochastic expression; random cell-division events and partitioning errors) is illustrated in Fig 2A and referred to as the full model. We also introduce two additional hybrid models [52, 53], where protein production and partitioning are considered in their deterministic limit (Fig 2B and 2C). Note that unlike the full model, where *x*(*t*) takes non-negative integer values, *x*(*t*) is continuous in the hybrid models. We will use these hybrid models for decomposing the protein noise level obtained from the full model into individual components representing contributions from different noise sources.

### Modeling the cell-cycle time

In order to quantify the steady-state protein mean and noise, we need to define the stochastic process that governs the timing of cell division. Variations in the duration of cell cycle can result from a variety of factors, such as cell physiology, growth rate, cell size and expression of genes that affect cell-cycle time such as FtsZ [24–32]. Given these complexities, we take a phenomenological approach to modeling cell-cycle time, and assume it to be an independent and identically distributed random variable that is drawn from a mixture of Erlang distributions (also known as phase-type distribution). The motivation for choosing this distribution is two fold:

1. Mixture of Erlang distributions can be represented via a continuous-time Markov chain, allowing mathematical tractability in terms of deriving/analyzing time evolution of moments.
2. This class of distribution is fairly general, in the sense that, any positively-valued distribution with *CV* ≤ 1 can be modeled via a mixture of Erlang distributions [54].

**Fig 3:**
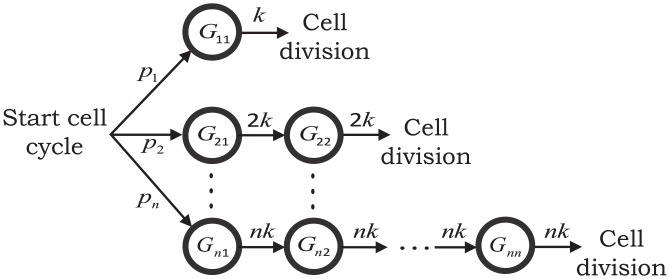
A continuous-time Markov chain model for the cell-cycle time. *Left*: The cell-cycle time is assumed to follow a mixture of Erlang distributions. At the start of cell cycle, a state *G*_*i*1_, *i* = {1, …, *n*} is chosen with probability *p*_*i*_. The cell cycle transitions through states *G*_*ij*_, *j* = {1, …, *i*} residing for an exponentially distributed time with mean 1/*ik* in each state. Cell division occurs after exit from *G*_*ii*_ and the above process is repeated.

Consider a mixture of *n* Erlang distributions with mixing probabilities *p*_*i*_, *i* = {1, …, *n*}. Recall that an Erlang distribution of order *i* is the distribution of the sum of *i* independent and identical exponential random variables. The cell-cycle time is assumed to have an Erlang distribution of order *i* with probability *p*_*i*_ and can be represented by a continuous-time Markov chain with states *G*_*ij*_, *j* = {1, …, *i*}, *i* = {1, …, *n*} (Fig 3). Let Bernoulli random variables *g*_*ij*_ = 1 if the system resides in state *G*_*ij*_ and 0 otherwise. The probability of transition *G*_*ij*_ → *G*_*i*(*j*+1)_ in the next infinitesimal time interval [*t*, *t* + *dt*) is given by *ikg*_*ij*_ *dt*, implying that the time spent in each state *G*_*ij*_ is exponentially distributed with mean 1/*ik*. To summarize, at the start of cell cycle, a state *G*_*i*1_, *i* = {1, …, *n*} is chosen with probability *p*_*i*_ and cell division occurs after transitioning through *i* exponentially distributed steps. Based on this formulation, the probability of a cell-division event occurring and a new cell cycle obtained from an Erlang distribution of size *i* starting in the next time interval [*t*, *t* + *dt*) is given by $kp_{i}\sum_{j=1}^{n}\left(jg_{jj}\right)dt$, and whenever the event occurs, the protein level changes as per Eq (4). Finally, the mean, the squared coefficient of variation, and the skewness of the cell-cycle time in terms of the Markov chain parameters are given by

$$\langle T\rangle =\frac{1}{k},\;CV_{T}^{2}=\sum_{i=1}^{n}\frac{p_{i}}{i},\;\text{Skewness}=\frac{\langle T^{3}\rangle -3\langle T\rangle \left(\langle T^{2}\rangle -{\langle T\rangle }^{2}\right)-{\langle T\rangle }^{3}}{{\left(\langle T^{2}\rangle -{\langle T\rangle }^{2}\right)}^{3/2}}=2\sum_{i=1}^{n}\frac{p_{i}}{i^{2}}$$ (7)

[55], where 〈*T*^3^〉 is the third-order moment of the cell-cycle time. An important property of this class of distributions is that increasing $CV_{T}^{2}$ also makes the distribution highly skewed, because from Eq (7) both the *CV* and skewness are linear combinations of *p*_*i*_, albeit with different linear coefficients that decrease with *i*. Considering that $\sum_{i=1}^{n}p_{i}=1$, the only way to increase $CV_{T}^{2}$ is by increasing smaller-index components and decreasing larger-index components of the distribution (i.e. increasing *p*_*i*_ and decreasing *p*_*j*_, where *i* < *j*). Since higher values of *i* are more penalized in the skewness equation, this would correspond to making the distribution more positively skewed. Hence high values of $CV_{T}^{2}$ also means high values of skewness, thus occurrences of longer cell cycles are more probable. As we will shortly see, this property leads to mean protein levels being dependent on $CV_{T}^{2}$.

## Results

### Computing the average number of protein molecules

All the models shown in Fig 2 are identical in terms of finding 〈*x*(*t*)〉 and in principle any one of them could have been used. We choose to analyze the full model illustrated in Fig 2A. Time evolution of the statistical moments of *x*(*t*) can be obtained from the Kolmogorov forward equations corresponding to the full model in Fig 2A combined with the cell-division process described in Fig 3. We refer the reader to [52, 56, 57] for an introduction to moment dynamics for stochastic and hybrid systems. Analysis in Appendix A in S1 Text shows

$$\frac{d\langle x\rangle }{dt}=k_{x}\langle B\rangle -\frac{k}{2}\sum_{j=1}^{n}\left(j\langle xg_{jj}\rangle \right).$$ (8)

Note that the time-derivative of the mean protein level (first-order moment) is unclosed, in the sense that, it depends on the second-order moment 〈*xg*_*ij*_〉. Typically, approximate closure methods are used to solve moments in such cases [52, 57–62]. However, the fact that *g*_*ij*_ is binary can be exploited to automatically close moment dynamics. In particular, since *g*_*ij*_ ∈ {0, 1}

$$\langle g_{ij}^{n}x^{m}\rangle =\langle g_{ij}x^{m}\rangle ,\;\;\;n\in \left\{1,2,\ldots \right\}$$ (9)

for any non-negative integer *m*. Moreover, as only a single state *g*_*ij*_ can be 1 at any time

$$\langle g_{ij}g_{rq}x^{m}\rangle =0,\;\text{if}\;i\neq r\;\text{or}\;j\neq q.$$ (10)

Using Eqs (9) and (10), the time evolutions of 〈*g*_*ij*_〉 and 〈*xg*_*ij*_〉 are obtained as

$$\frac{d\langle g_{i1}\rangle }{dt}=kp_{i}\sum_{j=1}^{n}\left(j\langle g_{jj}\rangle \right)-ik\langle g_{i1}\rangle ,$$ (11)

$$\frac{d\langle g_{ij}\rangle }{dt}=ik\langle g_{i(j-1)}\rangle -ik\langle g_{ij}\rangle ,\;j=\{2,\ldots ,i\},$$ (12)

$$\frac{d\langle xg_{i1}\rangle }{dt}=k_{x}\langle B\rangle \langle g_{i1}\rangle +\frac{k}{2}p_{i}\sum_{j=1}^{n}\left(j\langle xg_{jj}\rangle \right)-ik\langle xg_{i1}\rangle ,$$ (13)

$$\frac{d\langle xg_{ij}\rangle }{dt}=k_{x}\langle B\rangle \langle g_{ij}\rangle -ik\langle xg_{ij}\rangle +ik\langle xg_{i(j-1)}\rangle ,\;j=\{2,\ldots ,i\}$$ (14)

and only depend on 〈*g*_*ij*_〉 and 〈*xg*_*ij*_〉 (see Appendix A in S1 Text). Thus, Eqs (8) and (11)–(14) constitute a closed system of linear differential equations from which moments can be computed exactly.

To obtain an analytical formula for the average number of proteins, we start by performing a steady-state analysis of Eq (8) that yields

$$\begin{array}{c} \sum_{j=1}^{n}\left(j\bar{\langle xg_{jj}\rangle }\right)=\frac{2k_{x}\langle B\rangle }{k}, \end{array}$$ (15)

where $\bar{\langle .\rangle }$ denotes the expected value in the limit *t* → ∞. Using Eq (15), $\bar{\langle xg_{i1}\rangle }$ is determined from Eq (13), and then all moments $\bar{\langle xg_{ij}\rangle }$ are obtained recursively by performing a steady-state analysis of Eq (14) for *j* = {2, …, *i*}. This analysis results in

$$\begin{array}{c} \bar{\langle xg_{ij}\rangle }=\frac{k_{x}\langle B\rangle }{ik}p_{i}\left(1+\frac{j}{i}\right). \end{array}$$ (16)

Using Eqs (7), (16) and the fact that $\sum_{i=1}^{n}\sum_{j=1}^{i}g_{ij}=1$ we obtain the following expression for the mean protein level

$$\bar{\langle x\rangle }=\bar{\langle x\sum_{i=1}^{n}\sum_{j=1}^{i}g_{ij}\rangle }=\sum_{i=1}^{n}\sum_{j=1}^{i}\bar{\langle xg_{ij}\rangle }=\frac{k_{x}\langle B\rangle \langle T\rangle \left(3+CV_{T}^{2}\right)}{2}.$$ (17)

It is important to point that Eq (17) holds irrespective of the complexity, i.e., the number of states *G*_*ij*_ used in the phase-type distribution to approximate the cell-cycle time distribution. As expected, $\bar{\langle x\rangle }$ increases linearly with the average cell-cycle time duration 〈*T*〉 with longer cell cycles resulting in more accumulation of proteins. Consistent with previous findings, Eq (17) shows that the mean protein level is also affected by the randomness in the cell-cycle times $\left(CV_{T}^{2}\right)$ [40, 63]. For example, $\bar{\langle x\rangle }$ reduces by 25% as *T* changes from being exponentially distributed $\left(CV_{T}^{2}=1\right)$ to periodic $\left(CV_{T}^{2}=0\right)$ for fixed 〈*T*〉. Next, we determine the noise in protein copy numbers, as quantified by the squared coefficient of variation.

### Computing the protein noise level

Recall that the full model introduced in Fig 2A has three distinct noise mechanisms. Our strategy for computing the protein noise level is to first analyze the model with a single noise source, and then consider models with two and three sources. As shown below, this approach provides a systematic dissection of the protein noise level into components representing contributions from different mechanisms.

#### Contribution from randomness in cell-cycle times

We begin with the model shown in Fig 2B, where noise comes from a single source—random cell-division events. For this model, the time evolution of the second-order moment of the protein copy number is obtained as

$$\frac{d\langle x^{2}\rangle }{dt}=2k_{x}\langle B\rangle \langle x\rangle -\frac{3k}{4}\sum_{j=1}^{n}\left(j\langle x^{2}g_{jj}\rangle \right),$$ (18)

and depends on third-order moments 〈*x*^2^ *g*_*jj*_〉 (see Appendix B in S1 Text). Using the approach introduced earlier for obtaining the mean protein level, we close moment equations by writing the time evolution of moments 〈*x*^2^ *g*_*ij*_〉. Using Eqs (9) and (10)

$$\frac{d\langle x^{2}g_{i1}\rangle }{dt}=2k_{x}\langle B\rangle \langle xg_{i1}\rangle +\frac{k}{4}p_{i}\overset{n}{\underset{j=1}{\sum }}\left(j\langle x^{2}g_{jj}\rangle \right)-ik\langle x^{2}g_{i1}\rangle ,$$ (19)

$$\frac{d\langle x^{2}g_{ij}\rangle }{dt}=2k_{x}\langle B\rangle \langle xg_{ij}\rangle -ik\langle x^{2}g_{ij}\rangle +ik\langle x^{2}g_{\left(i-1\right)j}\rangle ,\;j=\left\{2,\ldots ,i\right\}.$$ (20)

Note that the moment dynamics for 〈*x*〉 and 〈*xg*_*ij*_〉 obtained in the previous section (Eqs (8), (13) and (14)) are identical for all the models in Fig 2, irrespective of whether the noise mechanism is modeled deterministically or stochastically. Eqs (8), (11)–(14) and (18)–(20) represent a closed set of linear differential equations and their steady-state analysis yields

$$\bar{\langle x^{2}g_{ij}\rangle }=\frac{k_{x}^{2}{\langle B\rangle }^{2}\langle T\rangle \left(3+CV_{T}^{2}\right)}{3ik}p_{i}+\frac{2k_{x}^{2}{\langle B\rangle }^{2}}{i^{2}k^{2}}\left(\frac{j^{2}+2ij+j}{2i}\right)p_{i}.$$ (21)

From Eq (21)

$$\bar{\langle x^{2}\rangle }=\bar{\langle x^{2}\sum_{i=1}^{n}\sum_{j=1}^{i}g_{ij}\rangle }=\sum_{i=1}^{n}\sum_{j=1}^{i}\bar{\langle x^{2}g_{ij}\rangle }=k_{x}^{2}{\langle B\rangle }^{2}\frac{\langle T^{3}\rangle +4CV_{T}^{2}{\langle T\rangle }^{3}+6{\langle T\rangle }^{3}}{3\langle T\rangle },$$ (22)

$$\langle T^{3}\rangle =\left(1+3CV_{T}^{2}+2\overset{n}{\underset{i=1}{\sum }}\frac{p_{i}}{i^{2}}\right){\langle T\rangle }^{3}.$$ (23)

Using Eq (22) and the mean protein count quantified in Eq (17), we obtain the following squared coefficient of variation

$$CV_{E}^{2}=\frac{1}{27}+\frac{4\left(9\frac{\langle T^{3}\rangle }{{\langle T\rangle }^{3}}-9-6CV_{T}^{2}-7CV_{T}^{4}\right)}{27{\left(3+CV_{T}^{2}\right)}^{2}},$$ (24)

where $CV_{E}^{2}$ represents the noise contribution from random cell-division events. Since cell division is a global event that affects expression of all genes, this noise contribution can also be referred to as *extrinsic noise* [49, 64–67]. In reality, there would be other sources of extrinsic noise, such as, fluctuations in the gene-expression machinery that we have ignored in this analysis.

Note that $CV_{E}^{2}\to 1/27$ as *T* approaches a delta distribution, i.e., cell divisions occur at fixed time intervals. We discuss simplifications of Eq (24) in various limits. For example, if the time taken to complete cell cycle is lognormally distributed, then

$$\frac{\langle T^{3}\rangle }{{\langle T\rangle }^{3}}={\left(1+CV_{T}^{2}\right)}^{3}\Rightarrow CV_{E}^{2}=\frac{1}{27}+\frac{4\left(21CV_{T}^{2}+20CV_{T}^{4}+9CV_{T}^{6}\right)}{27{\left(3+CV_{T}^{2}\right)}^{2}}$$ (25)

and extrinsic noise monotonically increases with $CV_{T}^{2}$. If fluctuations in *T* around 〈*T*〉 are small, then using Taylor series

$$\langle T^{3}\rangle /{\langle T\rangle }^{3}\approx 1+3CV_{T}^{2}.$$ (26)

Substituting Eq (26) in Eq (24) and ignoring $CV_{T}^{4}$ and higher order terms yields

$$CV_{E}^{2}\approx \frac{1}{27}+\frac{28CV_{T}^{2}}{81},$$ (27)

where the first term is the extrinsic noise for $CV_{T}^{2}\to 0$ and the second term is the additional noise due to random cell-division events.

#### Contribution from partitioning errors

Next, we consider the model illustrated in Fig 2C with both random cell-division events and partitioning of protein between the two daughter cells. Thus, the protein noise level here represents the contribution from both these sources. Analysis in Appendix C in S1 Text shows that the time evolution of 〈*x*^2^〉 and 〈*x*^2^ *g*_*ij*_〉 are given by

$$\frac{d\langle x^{2}\rangle }{dt}=2k_{x}\langle B\rangle \langle x\rangle +\frac{k}{4}\alpha \sum_{j=1}^{n}\left(j\langle xg_{jj}\rangle \right)-\frac{3k}{4}\sum_{j=1}^{n}\left(j\langle x^{2}g_{jj}\rangle \right),$$ (28)

$$\frac{d\langle x^{2}g_{i1}\rangle }{dt}=2k_{x}\langle B\rangle \langle xg_{i1}\rangle +\frac{k}{4}p_{i}\sum_{j=1}^{n}\left(j\langle x^{2}g_{jj}\rangle \right)+\frac{k}{4}\alpha p_{i}\sum_{j=1}^{n}\left(j\langle xg_{jj}\rangle \right)-ik\langle x^{2}g_{i1}\rangle ,$$ (29)

$$\frac{d\langle x^{2}g_{i1}\rangle }{dt}=2k_{x}\langle B\rangle \langle xg_{ij}\rangle -ik\langle x^{2}g_{ij}\rangle +ik\langle x^{2}g_{(i-1)j}\rangle ,\;j=\left\{2,\ldots ,i\right\}.$$ (30)

Note that Eqs (28) and (29) are slightly different from their counterparts obtained in the previous section (Eqs (18) and (19)) with additional terms that depend on *α*, where *α* quantifies the degree of partitioning error as defined in Eq (5). As expected, Eqs (28) and (29) reduce to Eqs (18) and (19) when *α* = 0 (i.e., deterministic partitioning). Computing $\bar{\langle x^{2}g_{ij}\rangle }$ by performing a steady-state analysis of Eqs (28)–(30) and using a similar approach as in Eq (22) we obtain

$$\bar{\langle x^{2}\rangle }=k_{x}^{2}{\langle B\rangle }^{2}\frac{\langle T^{3}\rangle +4CV_{T}^{2}{\langle T\rangle }^{3}+6{\langle T\rangle }^{3}}{3\langle T\rangle }+\frac{2\alpha k_{x}\langle B\rangle \langle T\rangle }{3}.$$ (31)

Finding *CV*^2^ of the protein level and subtracting the extrinsic noise found in Eq (24) yields

$$CV_{R}^{2}=\frac{4\alpha }{3(3+CV_{T}^{2})}\frac{1}{\bar{\langle x\rangle }},$$ (32)

where $CV_{R}^{2}$ represents the contribution of partitioning errors to the protein noise level. Intriguingly, while $CV_{R}^{2}$ increases with *α*, it decrease with $CV_{T}^{2}$. Thus, as cell-division times become more random for a fixed 〈*T*〉 and $\bar{\langle x\rangle }$, the noise contribution from partitioning errors decrease. It turns out that this dependence of $CV_{R}^{2}$ on *CV*_*T*_ is a direct result of the second equation in Eq (5), where stochasticity in the partitioning process increases linearly with *x*(*t*_*s*_), the number of protein molecules just before division. Based on Eq (17), one needs to reduce *k*_*x*_ or 〈*B*〉 to maintain a fixed $\bar{\langle x\rangle }$ for increasing randomness in cell-division times. Since the average number of protein molecules just before division is 2*k*_*x*_〈*B*〉〈*T*〉 (see Appendix D in S1 Text), a reduction in *k*_*x*_ or 〈*B*〉 results in a lower number of protein molecules before division, and hence, lesser noise from partitioning as per Eq (5) and a smaller $CV_{R}^{2}$. This reasoning is supported by the fact that if we redefine the noise in the partitioning process to make it independent of *x*(*t*_*s*_), i.e. modify Eq (5) as

$$\langle x_{+}\left(t_{s}\right)|x\left(t_{s}\right)\rangle =\frac{x(t_{s})}{2},\;\;\;\langle x_{+}^{2}\left(t_{s}\right)-{\langle x_{+}\left(t_{s}\right)\rangle }^{2}|x\left(t_{s}\right)\rangle =\alpha ,$$ (33)

then the noise contribution from partitioning errors is given by

$$CV_{R}^{2}=\frac{4\alpha }{3}\frac{1}{{\bar{\langle x\rangle }}^{2}},$$ (34)

and the dependency of $CV_{R}^{2}$ on *CV*_*T*_ disappears (Appendix D in S1 Text).

#### Contribution from stochastic expression

Finally, we consider the full model in Fig 2A with all the three different noise sources. For this model, moment dynamics is obtained as (see Appendix E in S1 Text)

$$\frac{d\langle x^{2}\rangle }{dt}=k_{x}\langle B^{2}\rangle +2k_{x}\langle B\rangle \langle x\rangle +\frac{k}{4}\alpha \sum_{j=1}^{n}\left(j\langle xg_{jj}\rangle \right)-\frac{3k}{4}\sum_{j=1}^{n}\left(j\langle x^{2}g_{jj}\rangle \right),$$ (35)

$$\frac{d\langle x^{2}g_{i1}\rangle }{dt}=k_{x}\langle B^{2}\rangle \langle g_{i1}\rangle +2k_{x}\langle B\rangle \langle xg_{i1}\rangle +\frac{k}{4}p_{i}\sum_{j=1}^{n}\left(j\langle x^{2}g_{jj}\rangle \right)+\frac{k}{4}\alpha p_{i}\sum_{j=1}^{n}\left(j\langle xg_{jj}\rangle \right)-ik\langle x^{2}g_{i1}\rangle ,$$ (36)

$$\frac{d\langle x^{2}g_{ij}\rangle }{dt}=k_{x}\langle B^{2}\rangle \langle g_{ij}\rangle +2k_{x}\langle B\rangle \langle xg_{ij}\rangle -ik\langle x^{2}g_{ij}\rangle +ik\langle x^{2}g_{(i-1)j}\rangle ,\;j=\left\{2,\ldots ,i\right\}.$$ (37)

Compared to Eqs (28)–(30), (35)–(37) have additional terms of the form *k*_*x*_〈*B*^2^〉, where 〈*B*^2^〉 is the second-order moment of the protein burst size in Eq (1). Performing an identical analysis as before we obtain

$$\bar{\langle x^{2}\rangle }=k_{x}^{2}{\langle B\rangle }^{2}\frac{\langle T^{3}\rangle +4CV_{T}^{2}{\langle T\rangle }^{3}+6{\langle T\rangle }^{3}}{3\langle T\rangle }+\frac{2\alpha k_{x}\langle B\rangle \langle T\rangle }{3}+\frac{k_{x}\langle B^{2}\rangle \langle T\rangle \left(3CV_{T}^{2}+5\right)}{2},$$ (38)

which yields the following total protein noise level

$$CV^{2}\;=\;CV_{E}^{2}+CV_{R}^{2}+CV_{P}^{2}=CV_{E}^{2}+\underset{\text{Intrinsic noise}}{\underset{︸}{\overset{\text{Partitioning noise }\left(CV_{R}^{2}\right)}{\overset{︷}{\frac{4a}{3\left(3+CV_{T}^{2}\right)}\frac{1}{\bar{\langle x\rangle }}}}\;+\;\overset{\text{Production noise }\left(CV_{P}^{2}\right)}{\overset{︷}{\frac{3CV_{T}^{2}+5}{3\left(3+CV_{T}^{2}\right)}\frac{\langle B^{2}\rangle }{\langle B\rangle }\frac{1}{\bar{\langle x\rangle }}}}}}$$ (39)

that can be decomposed into three terms. The first term $CV_{E}^{2}$ represents the contribution from random cell-division events and is given by Eq (24). The second term $CV_{R}^{2}$ is the contribution from partitioning errors determined in the previous section (partitioning noise), and the final term $CV_{P}^{2}$ is the additional noise representing the contribution from stochastic expression (production noise). A common approach to study gene expression noise is to decompose it into *intrinsic and extrinsic* components. These components are obtained experimentally using the dual-color assay that measures the correlation in the expression of two identical copies of the gene [49]. As per this definition, $CV_{E}^{2}$ represents the extrinsic noise as random cell-division events are common to all genes and makes expression levels more correlated in individual cells. In contrast, the contributions from noisy production and partitioning represent the intrinsic noise as they are specific to an individual gene and make expression levels less correlated.

An interesting observation from Eq (39) is that $CV_{T}^{2}$ has opposite effects on $CV_{R}^{2}$ and $CV_{P}^{2}$ (for fixed mean protein level). While $CV_{R}^{2}$ monotonically decreases with increasing $CV_{T}^{2}$, $CV_{P}^{2}$ increases with $CV_{T}^{2}$. Thus, if $\bar{\langle x\rangle }$ is small and *α* is large, then the noise contributed from partitioning dominates the total noise, and making cell-cycle duration more random will reduce the total noise. However, since both $CV_{E}^{2}$ and $CV_{P}^{2}$ are monotonically increasing functions of $CV_{T}^{2}$, the total noise will begin to increase with $CV_{T}^{2}$ once these noise sources become dominant. It turns out that in certain cases the intrinsic noise becomes invariant of $CV_{T}^{2}$. For example, when *B* = 1 with probability one, i.e., proteins are synthesized one at a time at exponentially distributed time intervals and *α* = 1 (binomial partitioning)

$$CV^{2}=CV_{E}^{2}+\frac{4}{3(3+CV_{T}^{2})}\frac{1}{\bar{\langle x\rangle }}+\frac{3CV_{T}^{2}+5}{3(3+CV_{T}^{2})}\frac{1}{\bar{\langle x\rangle }}=CV_{E}^{2}+\frac{1}{\bar{\langle x\rangle }}.$$ (40)

In this limit the intrinsic noise is always 1/Mean irrespective of the cell-cycle time distribution *T* [33]. Note that the average number of proteins itself depends on *T* as shown in Eq (17). Another important limit is $CV_{T}^{2}\to 0$, in which case Eq (39) reduces to

$$CV^{2}\approx \;\frac{\overset{CV_{E}^{2}}{\overset{︷}{1}}}{\underset{\text{Extrinsic noise}}{\underset{︸}{27}}}\;+\underset{\text{Intrinsic noise}}{\underset{︸}{\overset{CV_{R}^{2}}{\overset{︷}{\;\frac{4a}{9}\frac{1}{\bar{\langle x\rangle }}}}\;+\;\overset{CV_{P}^{2}}{\overset{︷}{\frac{5}{9}\frac{\langle B^{2}\rangle }{\langle B\rangle }\frac{1}{\bar{\langle x\rangle }}}}}},$$ (41)

and is similar to the result obtained in [38] for deterministic cell-division times and binomial partitioning.

**Fig 4:**
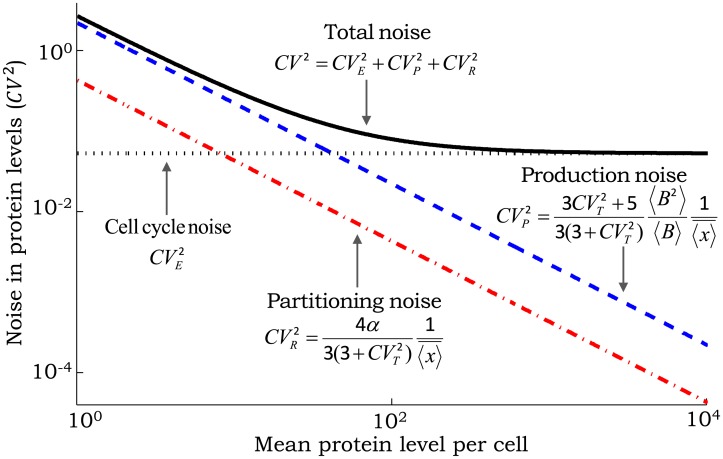
Scaling of noise as a function of the mean protein level for different mechanisms. The contribution of random cell-division events to the noise in protein copy numbers (extrinsic noise) is invariant of the mean. In contrast, contributions from partitioning errors at the time of cell division (partitioning noise) and stochastic expression (production noise) scale inversely with the mean. The scaling factors are shown as a function of the protein random burst size *B*, noise in cell-cycle time ($CV_{T}^{2}$) and magnitude of partitioning errors quantified by *α* (see Eq (5)). With increasing mean level the total noise first decreases and then reaches a baseline that corresponds to extrinsic noise. For this plot, *B* is assumed to be geometrically-distributed with mean 〈*B*〉 = 1.5, $CV_{T}^{2}=0.05$ and *α* = 1 (i.e., binomial partitioning).

Fig 4 shows how different protein noise components change as a function of the mean protein level as the gene’s transcription rate *k*_*x*_ is modulated. The extrinsic noise is primarily determined by the distribution of the cell-cycle time and is completely independent of the mean. In contrast, both $CV_{R}^{2}$ and $CV_{P}^{2}$ scale inversely with the mean, albeit with different scaling factors (Fig 4). This observation is particularly important since many single-cell studies in *E. coli*, yeast and mammalian cells have found the protein noise levels to scale inversely with the mean across different genes [68–71]. Based on this scaling it is often assumed that the observed cell-to-cell variability in protein copy numbers is a result of stochastic expression. However, as our results show, noise generated thorough partitioning errors is also consistent with these experimental observations and it may be impossible to distinguish between these two noise mechanisms based on protein *CV*^2^ versus mean plots unless *α* is known.

### Quantifying the effects of gene duplication on protein noise

**Fig 5:**
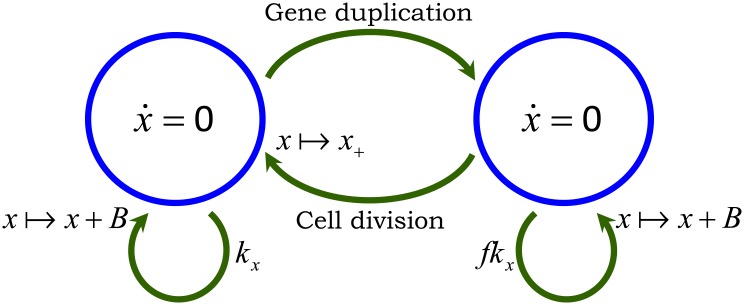
Model illustrating stochastic expression together with random gene-duplication and cell-division events. At the start of cell cycle, protein production occurs in stochastic bursts with rate *k*_*x*_. Genome duplication occurs at a random point *T*_1_ within the cell cycle and increases the burst arrival rate to *fk*_*x*_ (*f* > 1). Cell division occurs after time *T*_2_ from genome duplication, at which point the burst arrival rate reverts back to *k*_*x*_ and proteins are randomly partitioned between cells based on Eq (4).

The full model introduced in Fig 2 assumes that the transcription rate (i.e., the protein burst arrival rate) is constant throughout the cell cycle. This model is now extended to incorporate gene duplication during cell cycle, which increases the burst arrival (transcription) rate by *f* times (Fig 5). Note that due to gene dosage compensation, doubling the genome does not always correspond to *f* = 2 [72–74]. If *f* > 1, then accumulation of proteins will be bilinear as illustrated in Fig 1. As before, we again take a phenomenological approach to model the timing of gene duplication. The cell-cycle time *T* is divided into two intervals: time from the start of cell cycle to gene duplication (*T*_1_), and time from gene duplication to cell division (*T*_2_). *T*_1_ and *T*_2_ are independent random variables, each drawn from a mixture of Erlang distributions (see Fig. B in S1 Text). The mean cell-cycle duration and its noise can be expressed as

$$\begin{array}{ccc} & \langle T\rangle =\langle T_{1}\rangle +\langle T_{2}\rangle ,\;\;\beta =\frac{\langle T_{1}\rangle }{\langle T\rangle }, & CV_{T}^{2}=\beta^{2}CV_{T_{1}}^{2}+{\left(1-\beta \right)}^{2}CV_{T_{2}}^{2}, \end{array}$$ (42)

where $CV_{X}^{2}$ denotes the squared coefficient of variation of the random variable *X*. An important variable in this formulation is *β*, which represents the average time of gene duplication normalized by the mean cell-cycle time. Thus, *β* values close to 0 (1) imply that the gene is duplicated early (late) in the cell-cycle process. Moreover, the noise in the gene-duplication time is controlled via $CV_{T_{1}}^{2}$.

We refer the reader to Appendix F in S1 Text for a detailed analysis of the model in Fig 5 and only present the main results on the protein mean and noise levels. The steady-state mean protein count is given by

$$\begin{array}{cc} & \bar{\langle x\rangle }=\\ & \frac{k_{x}\langle B\rangle \langle T_{1}\rangle \left(2f\left(1-\beta \right)+3\beta +\beta CV_{T_{1}}^{2}\right)}{2}+\frac{k_{x}\langle B\rangle \langle T_{2}\rangle \left(3f\left(1-\beta \right)+4\beta +f\left(1-\beta \right)CV_{T_{2}}^{2}\right)}{2}, \end{array}$$ (43)

and decreases with *β*, i.e., a gene that duplicates early has on average, more number of proteins. When *β* = 1, then the transcription rate is *k*_*x*_ throughout the cell cycle and we recover the mean protein level obtained in Eq (17). Similarly, when *β* = 0 the transcription rate is *fk*_*x*_ and we obtain *f* times of the amount as in Eq (17). As per our earlier observation, more randomness in the timing of genome duplication and cell division (i.e., higher $CV_{T_{1}}^{2}$ and $CV_{T_{2}}^{2}$ values) increases $\bar{\langle x\rangle }$.

Our analysis shows that the total protein noise level can be decomposed into three components

$$CV^{2}=CV_{E}^{2}+CV_{R}^{2}+CV_{P}^{2}$$ (44)

where $CV_{E}^{2}$ is the extrinsic noise from random genome-duplication/cell-division events, and the sum of the contributions from partitioning errors ($CV_{R}^{2}$) and stochastic expression ($CV_{P}^{2}$) is the intrinsic noise. We refer the reader to Appendix F in S1 Text for noise formulas for any *f*, and only present formulas for *f* = 2 here. In this case, the intrinsic noise is obtained as

$$\begin{array}{cc} CV_{R}^{2}+CV_{P}^{2}= & \overset{CV_{R}^{2}}{\overset{︷}{\frac{4\alpha (2-\beta )}{3\left(\left(\beta^{2}-4\beta +6\right)+\beta^{2}CV_{T_{1}}^{2}+2{\left(1-\beta \right)}^{2}CV_{T_{2}}^{2}\right)}\frac{1}{\bar{\langle x\rangle }}}}\\ & +\overset{CV_{P}^{2}}{\overset{︷}{\frac{\left(10-8\beta +3\beta^{2}\right)+6{\left(1-\beta \right)}^{2}CV_{T_{2}}^{2}+3\beta^{2}CV_{T_{1}}^{2}}{3\left(\left(\beta^{2}-4\beta +6\right)+\beta^{2}CV_{T_{1}}^{2}+2{\left(1-\beta \right)}^{2}CV_{T_{2}}^{2}\right)}\frac{\langle B^{2}\rangle }{\langle B\rangle }\frac{1}{\bar{\langle x\rangle }}}}. \end{array}$$ (45)

Note that for *β* = 0 and 1, we recover the intrinsic noise level in Eq (39) from Eq (45). Interestingly, for *B* = 1 with probability 1 and *α* = 1, the intrinsic noise is always 1/Mean irrespective of the values chosen for $CV_{T_{1}}^{2}$, $CV_{T_{2}}^{2}$ and *β*. For high precision in the timing of cell-cycle events (*CV*_*T*_1__ → 0, *CV*_*T*_2__ → 0)

$$CV^{2}\approx \;\frac{\overset{CV_{E}^{2}}{\overset{︷}{4-3{\left(\beta -2\right)}^{2}\beta^{2}}}}{\underset{\text{Extrinsic noise}}{\underset{︸}{3{\left(\beta^{2}-4\beta +6\right)}^{2}}}}\;+\;\underset{\text{Intrinsic noise}}{\underset{︸}{\overset{CV_{R}^{2}}{\overset{︷}{\frac{4\alpha \left(2-\beta \right)}{3\left(\beta^{2}-4\beta +6\right)}\frac{1}{\bar{\langle x\rangle }}}}\;+\overset{CV_{P}^{2}}{\overset{︷}{\;\frac{\left(10-8\beta +3\beta^{2}\right)}{3\left(\beta^{2}-4\beta +6\right)}\frac{\langle B^{2}\rangle }{\langle B\rangle }\frac{1}{\bar{\langle x\rangle }}}}}},$$ (46)

where mean protein level is given by

$$\bar{\langle x\rangle }\approx \frac{k_{x}\langle B\rangle \langle T_{1}\rangle \left(4-\beta \right)}{2}+k_{x}\langle B\rangle \langle T_{2}\rangle \left(3-\beta \right).$$ (47)

**Fig 6:**
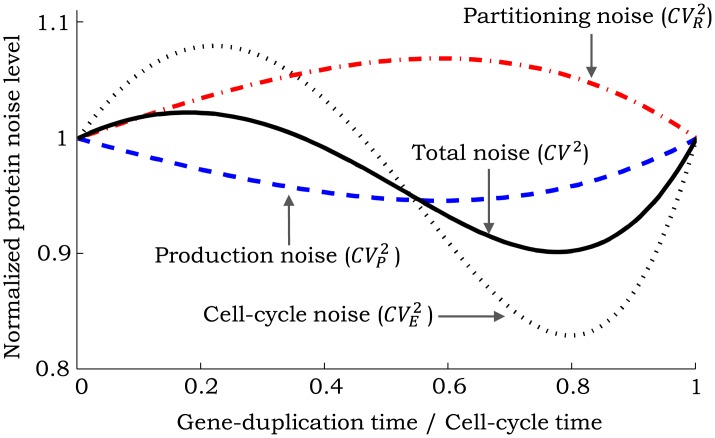
Contributions from different noise sources as a function of the timing of genome duplication for $CV_{T_{1}}^{2}=CV_{T_{2}}^{2}=0.05$. Different noise components in Eq (46) are plotted as a function of *β*, which represents the fraction of time within the cell cycle at which gene duplication occurs. The mean protein level is held constant by simultaneously changing the transcription rate *k*_*x*_. Noise levels are normalized by their respective value at *β* = 0. The noise contribution from partitioning errors is maximized at *β* ≈ 0.6. In contrast, the contribution from stochastic expression is minimum at *β* ≈ 0.6. The extrinsic noise contribution from random gene-duplication and cell-division events is maximum at *β* ≈ 0.2 and minimum at *β* ≈ 0.8. For this plot, the mean of the protein is 170 molecules per cell; and the bursts are geometrically distributed with 〈*B*〉 = 10.

We investigate how different noise components in Eq (46) vary with *β* as the mean protein level is held fixed by changing *k*_*x*_. Fig 6 shows that $CV_{P}^{2}$ follows a U-shaped profile with the optima occurring at $\beta =2-\sqrt{2}\approx 0.6$ and the corresponding minimum value being ≈ 5% lower than its value at *β* = 0. An implication of this result is that if stochastic expression is the dominant noise source, then gene duplication can result in slightly lower protein noise levels. In contrast to $CV_{P}^{2}$, $CV_{R}^{2}$ has a maxima at $\beta =2-\sqrt{2}$ which is ≈ 6% higher than its value at *β* = 0 (Fig 6). Analysis in Appendix F5 in S1 Text reveals that $CV_{R}^{2}$ and $CV_{P}^{2}$ follow the same qualitative shapes as in Fig 6 for any $CV_{T_{1}}^{2}$ and $CV_{T_{2}}^{2}$. Interestingly, when $CV_{T_{1}}^{2}=CV_{T_{2}}^{2}$, the maximum and minimum values of $CV_{R}^{2}$ and $CV_{P}^{2}$ always occur at $\beta =2-\sqrt{2}$ albeit with different optimal values than Fig 6 (see Fig. C in S1 Text). For example, if $CV_{T_{1}}^{2}=CV_{T_{2}}^{2}=1$ (i.e., exponentially distributed *T*_1_ and *T*_2_), then the maximum value of $CV_{R}^{2}$ is 20% higher and the minimum value of $CV_{P}^{2}$ is 10% lower than their respective value for *β* = 0. Given that the effect of changing *β* on $CV_{P}^{2}$ and $CV_{R}^{2}$ is small and antagonistic, the overall affect of genome duplication on intrinsic noise may be minimal and hard to detect experimentally.

While the above analysis is for a stable protein, a natural question to ask is how would these results change for an unstable protein? Consider an unstable protein with half-life considerably shorter than the cell-cycle duration. This rapid turnover ensures that the protein level equilibrates instantaneously after cell-division and gene-duplication events. Let *γ*_*x*_ denote the protein decay rate. Then, the mean protein level before and after genome duplication is $\bar{\langle x\rangle }=k_{x}\langle B\rangle /\gamma_{x}$ and $\bar{\langle x\rangle }=2k_{x}\langle B\rangle /\gamma_{x}$, respectively. Note that in the limit of large *γ*_*x*_ there is no noise contribution from partitioning errors since errors incurred at the time of cell division would be instantaneously corrected. The extrinsic noise, which can be interpreted as the protein noise level for deterministic protein production and decay is obtained as (for analysis on general *f* see Appendix G in S1 Text)

$$CV_{E}^{2}=\frac{(1-\beta )\beta }{{\left(2-\beta \right)}^{2}},$$ (48)

which is similar to noise level reported in [75]. When *β* = 0 or 1, the transcription rate and the protein level are constant within the cell cycle and $CV_{E}^{2}=0$. Moreover, $CV_{E}^{2}$ is maximized at *β* = 2/3 with a value of 1/8. Thus, in contrast to a stable protein, extrinsic noise in an unstable protein is strongly dependent on the timing of gene duplication. Next, consider the intrinsic noise component. Analysis in Appendix G in S1 Text shows that the noise contribution from random protein production and decay is

$$CV_{P}^{2}=\frac{1}{2}\left(\frac{\langle B^{2}\rangle }{\langle B\rangle }+1\right)\frac{1}{\bar{\langle x\rangle }},\;\;\bar{\langle x\rangle }=\frac{k_{x}\langle B\rangle \left(2-\beta \right)}{\gamma_{x}}.$$ (49)

While the mean protein level is strongly dependent on *β*, the intrinsic noise Fano factor $=CV_{P}^{2}\times \langle x\rangle$ is independent of it. Thus, similar to what was observed for a stable protein, the intrinsic noise in an unstable protein is invariant of *β* for a fixed $\bar{\langle x\rangle }$.

## Discussion

We have investigated a model of protein expression in bursts coupled to discrete gene-duplication and cell-division events. The novelty of our modeling framework lies in describing the size of protein bursts *B*, the time between cell birth and gene duplication *T*_1_, the time between gene duplication and cell division *T*_2_, and partitioning of molecules during cell division through general statistical distributions. Exact formulas connecting the protein mean and noise levels to these underlying distributions were derived. Furthermore, the protein noise level, as measured by the squared coefficient of variation, was decomposed into three components representing contributions from gene-duplication/cell-division events, stochastic expression and random partitioning. While the first component is independent of the mean protein level, the other two components are inversely proportional to it. Some important insights are as follows:

- The mean protein level is affected by both the first and second-order moments of *T*_1_ and *T*_2_. In particular, randomness in these times (for a fixed mean) increases the average protein count. This increase can be attributed to the fact that increasing cell-cycle time variations leads to positively skewed distributions, making longer cell cycles (and hence higher protein accumulation) more likely.
- Random gene-duplication/cell-division events create an extrinsic noise term which is completely determined by moments of *T*_1_ and *T*_2_ up to order three. Interestingly, noise in the timing of these events also critically affects the intrinsic noise contributions from stochastic expression and partitioning. Hence, ignoring the effect of cell-cycle time variations, may lead to erroneous estimation of intrinsic noise.
- The noise contribution from partitioning errors decreases with increasing randomness in *T*_1_ and *T*_2_. Thus, if $\bar{\langle x\rangle }$ is sufficiently small and *α* is large compared to *B* in Eq (45), increasing noise in the timing of cell-cycle events decreases the total noise level.

A key limitation of our approach is to model timing of gene-duplication/cell-division events through independent random variables. There is always non-zero correlation in the cell-cycle durations of mother and daughter cells [76–78]. Moreover, in the same cell cycle, times *T*_1_ and *T*_2_ could be dependent [79]. While our assumption on independence of timing maybe unrealistic, it played an important role in deriving exact analytical formulas for the protein mean and noise levels. We have used Monte Carlo simulations to investigate scenarios where *T*_1_ and *T*_2_, or successive cell-division events, have memory and are dependent random variables (see Appendix H in S1 Text). Our analysis reveals that the results presented in Figs 4 and 6 hold even when the assumption of independent timing is perturbed over biologically meaningful parameters.

### Effect of gene duplication on noise level

In this first-of-its-kind study, we have investigated how discrete *f*-fold changes in the transcription rate due to gene duplication affect the intercellular variability in protein levels. Not surprisingly, the timing of genome duplication strongly affects the mean protein level—$\bar{\langle x\rangle }$ can change up to *f* folds depending on whether the gene duplicates early (*β* = 0) or late (*β* = 1) in the cell cycle. Results show that genome duplication has counter intuitive effects on the protein noise level (Fig 6). For example, if stochastic expression is the dominant source of noise, then doubling of transcription due to duplication results in lower noise, as compared to constant transcription throughout the cell cycle. This is because for the same mean protein level, there are more burst (transcription) events in the case of genome duplication (*f* = 2) than constant transcription (*f* = 1). For example, consider deterministic timing ($CV_{T_{1}}^{2}=CV_{T_{2}}^{2}=0$) and gene duplication in the middle of the cell cycle (*β* = 0.5). Then, for the case *β* = 1, there are on average *k*_*x*_〈*T*〉 burst events per cell cycle. For the same $\bar{\langle x\rangle }$, there are 1.05*k*_*x*_〈*T*〉 production events in the case of gene duplication (*β* = 0.5). This slight increase in the number of transcription events leads to better averaging of bursty protein synthesis and lower noise levels. Overall, the effect of *β* on different noise component is quite modest: as *β* varies, noise components deviate at maximum ≈ 20% from their values at *β* = 0 (Fig 6). These results are in contrast to the case of an unstable protein, where noise from the cell-cycle process is strongly dependent on *β* as shown in Eq (48).

### Noise in synchronized cells

The mathematical framework introduced for modeling timing of cell division can be easily used to compute noise in synchronized cells. For example, let the cell-cycle duration be an Erlang distribution with shape parameter *n* and rate parameter *nk* (i.e., *p*_*n*_ = 1 in Fig 3), which can be biologically interpreted as cells moving through *n* cell-cycle stages *G*_*n*1_, *G*_*n*2_, …, *G*_*nn*_. Statistical moments conditioned on the cell-cycle stage *G*_*nj*_ can be obtained using

$$\bar{\langle x^{m}|g_{nj}=1\rangle }=\frac{\bar{\langle g_{nj}x^{m}\rangle }}{\bar{\langle g_{nj}\rangle }},\;\;m\in \left\{1,2\right\}.$$ (50)

Using Eq (50) and moments $\bar{\langle g_{nj}x^{m}\rangle }$ obtained from Eqs (16) and (35)–(37), yields the following conditional mean

$$\begin{array}{c} \bar{\langle x|g_{nj}=1\rangle }=k_{x}\langle B\rangle \langle T\rangle \left(1+\frac{j}{n}\right), \end{array}$$ (51)

which increases with cell-cycle stage (i.e., higher values of *j*). The protein noise level given that cells are in stage *G*_*nj*_ is given by

$$\begin{array}{lll} CV^{2}{|}_{g_{nj}=1} & ≔ & \frac{\bar{\langle x^{2}|g_{nj}=1\rangle }-{\bar{\langle x|g_{nj}=1\rangle }}^{2}}{{\bar{\langle x|g_{nj}=1\rangle }}^{2}}\\ & = & \underset{\text{Extrinsic noise}}{\underset{︸}{\overset{CV_{E}^{2}}{\overset{︷}{\frac{n+3j}{3{\left(n+j\right)}^{2}}}}}}+\underset{\text{Intrinsic noise}}{\underset{︸}{\overset{CV_{R}^{2}}{\overset{︷}{\frac{2n\alpha }{3(n+j)}\frac{1}{\bar{\langle x|g_{nj}\rangle }}}}+\overset{CV_{P}^{2}}{\overset{︷}{\frac{n+3j}{3(n+j)}\frac{\langle B^{2}\rangle }{\langle B\rangle }\frac{1}{\bar{\langle x|g_{nj}\rangle }}}}}}. \end{array}$$ (52)

Note that if *n* is large then the first term, which represents the noise contribution from the cell-cycle process, is negligible and can be dropped. Interesting, while the noise contribution from partitioning errors $CV_{R}^{2}$ decreases with cell-cycle stage, the noise contribution from stochastic expression $CV_{P}^{2}$ increases with *j*. Moreover, for *B* = 1 with probability 1 and *α* = 1, the intrinsic noise is always 1/Mean irrespective of *j*. Assuming high *n*, the noise at cell birth (*j* = 1) and division (*j* = *n*) are obtained as

$$CV^{2}{|}_{g_{n1}=1}=\underset{\text{Intrinsic noise}}{\underset{︸}{\overset{CV_{R}^{2}}{\overset{︷}{\frac{2\alpha }{3}\frac{1}{\bar{\langle x|g_{n1}\rangle }}}}+\overset{CV_{P}^{2}}{\overset{︷}{\frac{1}{3}\frac{\langle B^{2}\rangle }{\langle B\rangle }\frac{1}{\bar{\langle x|g_{n1}\rangle }}}}}}$$ (53)

$$CV^{2}{|}_{g_{nn}=1}=\underset{\text{Intrinsic noise}}{\underset{︸}{\overset{CV_{R}^{2}}{\overset{︷}{\frac{\alpha }{3}\frac{1}{\bar{\langle x|g_{nn}\rangle }}}}+\overset{CV_{P}^{2}}{\overset{︷}{\frac{2}{3}\frac{\langle B^{2}\rangle }{\langle B\rangle }\frac{1}{\bar{\langle x|g_{nn}\rangle }}}}}},$$ (54)

respectively. Thus, measurements of Eqs (53) and (54) by synchronizing cells (or by using cell size as a proxy for cell-cycle stage) can be used to quantify *α* and 〈*B*^2^〉/〈*B*〉, providing a novel way to separate these noise contributions. Next, we discuss how noise in asynchronous cell can be used to quantify these parameters.

### Parameter inference in asynchronous cells

Simple models of bursty expression and decay predict the distribution of protein levels to be negative binomial (or gamma distributed in the continuous framework) [80, 81]. These distributions are characterized by two parameter—the burst arrival rate *k*_*x*_ and the average burst size 〈*B*〉, which can be estimated from measured protein mean and noise levels. This method has been used for estimating *k*_*x*_ and 〈*B*〉 across different genes in *E. coli* [47, 82]. Our detailed model that takes into account partitioning errors predicts (ignoring gene-duplication effects)

$$\begin{array}{cc} \text{Intrinsic}\;\text{noise}= & \frac{4\alpha }{3(3+CV_{T}^{2})}\frac{1}{\bar{\langle x\rangle }}+\frac{3CV_{T}^{2}+5}{3(3+CV_{T}^{2})}\frac{\langle B^{2}\rangle }{\langle B\rangle }\frac{1}{\bar{\langle x\rangle }}. \end{array}$$ (55)

Using $CV_{T}^{2}⪡1$ and a geometrically distributed *B* [50, 83–85], Eq (55) reduces to

$$\begin{array}{cc} \text{Intrinsic}\;\text{noise}= & \frac{4\alpha }{9}\frac{1}{\bar{\langle x\rangle }}+\frac{5}{9}\frac{1+2\langle B\rangle }{\bar{\langle x\rangle }}. \end{array}$$ (56)

Given measurements of intrinsic noise and the mean protein level, 〈*B*〉 can be estimated from Eq (56) assuming *α* = 1 (i.e., binomial partitioning). Once 〈*B*〉 is known, *k*_*x*_ is obtained from the mean protein level given by Eq (17). Since for many genes 〈*B*〉 ≈ 0.5–5 [47], the contribution of the first term in Eq (56) is significant, and ignoring it could lead to overestimation of 〈*B*〉. Overestimation would be even more severe if *α* happen to be much higher than 1, as would be the case for proteins that form aggregates or multimers [33]. One approach to estimate both 〈*B*〉 and *α* is to measure intrinsic noise changes in response to perturbing 〈*B*〉 by, for example, changing the mRNA translation rate through mutations in the ribosomal-binding sites (RBS). Consider a hypothetical scenario where the Fano Factor (intrinsic noise times the mean level) is 6. Let mutations in the RBS reduces $\bar{\langle x\rangle }$ by 50%, implying a 50% reduction in 〈*B*〉. If the Fano factor changes from 6 to 4 due to this mutation, then 〈*B*〉 = 3.6 and 〈*α*〉 = 3.25.

Our recent work has shown that higher-order statistics of protein levels (i.e., skewness and kurtosis) or transient changes in protein noise levels in response to blocking transcription provide additional information for discriminating between noise mechanisms [86, 87]. Up till now these studies have ignored noise sources in the cell-cycle process. It remains to be seen if such methods can be used for separating the noise contributions of partitioning errors and stochastic expression to reliably estimate 〈*B*〉 and *α*.

### Integrating cell size and promoter switching

An important limitation of our modeling approach is that it does not take into account the size of growing cells. Recent experimental studies have provided important insights into the regulatory mechanisms controlling cell size [88–91]. More specifically, studies in *E. coli* and yeast argue for an “adder” model, where cell-cycle timing is controlled so as to add a constant volume between cell birth and division [78, 91–93]. Assuming exponential growth, this implies that the time taken to complete cell cycle is negatively correlated with cell size at birth. More importantly, cell size also affects gene expression—in mammalian cells transcription rates linearly increase with the cell size [94]. Thus, as cells become bigger they also produce more mRNAs to ensure gene product concentrations remains more or less constant. A main direction of future work would be to explicitly include cell size with size-dependent expression and timing of cell division determined by the adder model. This formulation will for the first time, allow simultaneous investigation of stochasticity in cell size, protein molecular count and concentration.

Our study ignores genetic promoter switching between active and inactive states, which has been shown to be a major source of noise in the expression of genes across organisms [95–104]. Taking into account promote switching is particularly important for genome-duplication studies, where doubling the number of gene copies could lead to more efficient averaging of promoter fluctuations. Another direction of future work will be to incorporate this additional noise source into the modeling framework and investigate its contribution as a function of gene-duplication timing.

## Supporting Information

S1 Text Supplementary Material. Additional proofs and simulations.
(PDF)

## References

[1] Blake WJ, Kaern M, Cantor CR, Collins JJ. Noise in eukaryotic gene expression. Nature. 2003;422:633–637. 10.1038/nature01546 12687005

[2] Raser JM, O’Shea EK. Noise in Gene Expression: Origins, Consequences, and Control. Science. 2005;309:2010–2013. 10.1126/science.1105891 16179466 PMC1360161

[3] Neuert G, Munsky B, Tan RZ, Teytelman L, Khammash M, van Oudenaarden A. Systematic Identification of Signal-Activated Stochastic Gene Regulation. Science. 2013;339:584–587. 10.1126/science.1231456 23372015 PMC3751578

[4] Libby E, Perkins TJ, Swain PS. Noisy information processing through transcriptional regulation. Proceedings of the National Academy of Sciences. 2007;104:7151–7156. 10.1073/pnas.0608963104 PMC1855426 17420464

[5] Fraser HB, Hirsh AE, Giaever G, Kumm J, Eisen MB. Noise Minimization in Eukaryotic Gene Expression. PLOS Biology. 2004;2:e137 10.1371/journal.pbio.0020137 15124029 PMC400249

[6] Lehner B. Selection to minimise noise in living systems and its implications for the evolution of gene expression. Molecular Systems Biology. 2008;4:170 10.1038/msb.2008.11 18319722 PMC2290932

[7] Losick R, Desplan C. Stochasticity and Cell Fate. Science. 2008;320:65–68. 10.1126/science.1147888 18388284 PMC2605794

[8] Arkin AP, Ross J, McAdams HH. Stochastic Kinetic Analysis of Developmental Pathway Bifurcation in Phage *λ*-Infected *Escherichia coli* Cells. Genetics. 1998;149:1633–1648. 9691025 10.1093/genetics/149.4.1633 PMC1460268

[9] Weinberger L, Burnett J, Toettcher J, Arkin A, Schaffer D. Stochastic Gene Expression in a Lentiviral Positive-Feedback Loop: HIV-1 Tat Fluctuations Drive Phenotypic Diversity. Cell. 2005;122:169–182. 10.1016/j.cell.2005.06.006 16051143

[10] Weinberger LS, Dar RD, Simpson ML. Transient-mediated fate determination in a transcriptional circuit of HIV. Nature Genetics. 2008;40:466–470. 10.1038/ng.116 18344999

[11] Singh A, Weinberger LS. Stochastic gene expression as a molecular switch for viral latency. Current Opinion in Microbiology. 2009;12:460–466. 10.1016/j.mib.2009.06.016 19595626 PMC2760832

[12] Dar RD, Hosmane NN, Arkin MR, Siliciano RF, Weinberger LS. Screening for noise in gene expression identifies drug synergies. Science. 2014;344:1392–1396. 10.1126/science.1250220 24903562 PMC4122234

[13] Eldar A, Elowitz MB. Functional roles for noise in genetic circuits. Nature. 2010;467:167–173. 10.1038/nature09326 20829787 PMC4100692

[14] Veening JW, Smits WK, Kuipers OP. Bistability, Epigenetics, and Bet-Hedging in Bacteria. Annual Review of Microbiology. 2008;62:193–210. 10.1146/annurev.micro.62.081307.163002 18537474

[15] Kussell E, Leibler S. Phenotypic diversity, population growth, and information in fluctuating environments. Science. 2005;309:2075–2078. 10.1126/science.1114383 16123265

[16] Balaban NQ, Merrin J, Chait R, Kowalik L, Leibler S. Bacterial persistence as a phenotypic switch. Science. 2004;305:1622–1625. 10.1126/science.1099390 15308767

[17] Sánchez-Romero MA, Casadesús J. Contribution of phenotypic heterogeneity to adaptive antibiotic resistance. Proceedings of the National Academy of Sciences. 2014;111:355–360. 10.1073/pnas.1316084111 PMC3890857 24351930

[18] Neildez-Nguyen TMA, Parisot A, Vignal C, Rameau P, Stockholm D, Picot J, et al Epigenetic gene expression noise and phenotypic diversification of clonal cell populations. Differentiation. 2008;76:33–40. 10.1111/j.1432-0436.2007.00219.x 17825084

[19] Paldi A. Stochastic gene expression during cell differentiation: order from disorder? Cellular and Molecular Life Sciences. 2003;60:1775–1778. 10.1007/s00018-003-23147-z 14523542 PMC11138758

[20] Raj A, van Oudenaarden A. Nature, nurture, or chance: stochastic gene expression and its consequences. Cell. 2008;135:216–226. 10.1016/j.cell.2008.09.050 18957198 PMC3118044

[21] Kaern M, Elston TC, Blake WJ, Collins JJ. Stochasticity in gene expression: from theories to phenotypes. Nature Reviews Genetics. 2005;6:451–464. 10.1038/nrg1615 15883588

[22] Magklara A, Lomvardas S. Stochastic gene expression in mammals: lessons from olfaction. Trends in Cell Biology. 2013;23:449–456. 10.1016/j.tcb.2013.04.005 23689023 PMC3755038

[23] Munsky B, Trinh B, Khammash M. Listening to the noise: random fluctuations reveal gene network parameters. Molecular systems biology. 2009;5:318 10.1038/msb.2009.75 19888213 PMC2779089

[24] Wang P, Robert L, Pelletier J, Dang WL, Taddei F, Wright A, et al Robust growth of *Escherichia coli*. Current biology. 2010;20:1099–1103. 10.1016/j.cub.2010.04.045 20537537 PMC2902570

[25] Lambert G, Kussell E. Quantifying Selective Pressures Driving Bacterial Evolution Using Lineage Analysis. Physical Review X. 2015;5:011016 10.1103/PhysRevX.5.011016 26213639 PMC4511495

[26] Tsukanov R, Reshes G, Carmon G, Fischer-Friedrich E, Gov NS, Fishov I, et al Timing of Z-ring localization in *Escherichia coli*. Physical Biology. 2011;8:066003 10.1088/1478-3975/8/6/066003 22015938

[27] Reshes G, Vanounou S, Fishov I, Feingold M. Cell shape dynamics in *Escherichia coli*. Biophysical Journal. 2008;94:251–264. 10.1529/biophysj.107.104398 17766333 PMC2134870

[28] Reshes G, Vanounou S, Fishov I, Feingold M. Timing the start of division in *E. coli*: a single-cell study. Physical Biology. 2008;5:046001 10.1088/1478-3975/5/4/046001 18997273

[29] Roeder AH, Chickarmane V, Chuna A, Obara B, Manjunath BS, Meyerowitz EM. Variability in the control of cell division underlies sepal epidermal patterning in Arabidopsis thaliana. PLOS Biology. 2010;8:e1000367 10.1371/journal.pbio.1000367 20485493 PMC2867943

[30] Zilman A, Ganusov VV, Perelson AS. Stochastic models of lymphocyte proliferation and death. PLOS ONE. 2010;5:e12775 10.1371/journal.pone.0012775 20941358 PMC2948000

[31] Hawkins ED, Markham JF, McGuinness LP, Hodgkin PD. A single-cell pedigree analysis of alternative stochastic lymphocyte fates. Proceedings of the National Academy of Sciences. 2009;106:13457–13462. 10.1073/pnas.0905629106 PMC2715326 19633185

[32] Stukalin EB, Aifuwa I, Kim JS, Wirtz D, Sun SX. Age-dependent stochastic models for understanding population fluctuations in continuously cultured cells. Journal of The Royal Society Interface. 2013;101 10.1098/rsif.2013.0325 PMC3971721 23760298

[33] Huh D, Paulsson J. Random partitioning of molecules at cell division. Proceedings of the National Academy of Sciences. 2011;108:15004–15009. 10.1073/pnas.1013171108 PMC3169110 21873252

[34] Gonze D. Modeling the effect of cell division on genetic oscillators. Journal of Theoretical Biology. 2013;325:22–33. 10.1016/j.jtbi.2013.02.001 23434891

[35] Lloyd-Price J, Tran H, Ribeiro AS. Dynamics of small genetic circuits subject to stochastic partitioning in cell division. Journal of Theoretical Biology. 2014;356:11–19. 10.1016/j.jtbi.2014.04.018 24768865

[36] Zopf CJ, Quinn K, Zeidman J, Maheshri N. Cell-Cycle Dependence of Transcription Dominates Noise in Gene Expression. PLOS Computational Biology. 2013;9:e1003161 10.1371/journal.pcbi.1003161 23935476 PMC3723585

[37] Narula J, Kuchina A, Lee DyD, Fujita M, Süel GM, Igoshin OA. Chromosomal Arrangement of Phosphorelay Genes Couples Sporulation and DNA Replication. Cell. 2015;162:328–337. 10.1016/j.cell.2015.06.012 26165942 PMC4506695

[38] Schwabe A, Bruggeman FJ. Contributions of Cell Growth and Biochemical Reactions to Nongenetic Variability of Cells. Biophysical Journal. 2014;107:301–313. 10.1016/j.bpj.2014.05.004 25028872 PMC4104058

[39] Huh D, Paulsson J. Non-genetic heterogeneity from stochastic partitioning at cell division. Nature Genetics. 2011;43:95–100. 10.1038/ng.729 21186354 PMC3208402

[40] Antunes D, Singh A. Quantifying gene expression variability arising from randomness in cell division times. Journal of Mathematical Biology. 2015; 71: 437–463. 10.1007/s00285-014-0811-x 25182129

[41] Yu J, Xiao J, Ren X, Lao K, Xie XS. Probing Gene Expression in Live Cells, One Protein Molecule at a Time. Science. 2006;311:1600–1603. 10.1126/science.1119623 16543458

[42] Paulsson J. Model of stochastic gene expression. Physics of Life Reviews. 2005;2:157–175. 10.1016/j.plrev.2005.03.003

[43] Shahrezaei V, Swain PS. Analytical distributions for stochastic gene expression. Proceedings of the National Academy of Sciences. 2008;105:17256–17261. 10.1073/pnas.0803850105 PMC2582303 18988743

[44] Singh A, Hespanha JP. Optimal Feedback Strength for Noise Suppression in Autoregulatory Gene Networks. Biophysical Journal. 2009;96:4013–4023. 10.1016/j.bpj.2009.02.064 19450473 PMC2712194

[45] Jia T, Kulkarni RV. Intrinsic Noise in Stochastic Models of Gene Expression with Molecular Memory and Bursting. Physical Review Letters. 2011; 106: 058102 10.1103/PhysRevLett.106.058102 21405439

[46] Alon U. An Introduction to Systems Biology: Design Principles of Biological Circuits. Chapman and Hall/CRC; 2006.

[47] Taniguchi Y, Choi PJ, Li GW, Chen H, Babu M, Hearn J, et al Quantifying *E. coli* proteome and transcriptome with single-molecule sensitivity in single cells. Science. 2010;329:533–538. 10.1126/science.1188308 20671182 PMC2922915

[48] Schwanhausser B, Busse D, Li N, Dittmar G, Schuchhardt J, Wolf J, et al Global quantification of mammalian gene expression control. Nature. 2011;473:337–342. 10.1038/nature10098 21593866

[49] Swain PS, Elowitz MB, Siggia ED. Intrinsic and extrinsic contributions to stochasticity in gene expression. Proceedings of the National Academy of Sciences. 2002;99:12795–12800. 10.1073/pnas.162041399 PMC130539 12237400

[50] Berg OG. A model for the statistical fluctuations of protein numbers in a microbial population. Journal of Theoretical Biology. 1978;71:587–603. 10.1016/0022-5193(78)90326-0 96307

[51] Rigney DR. Stochastic model of constitutive protein levels in growing and dividing bacterial cells. Journal of Theoretical Biology. 1979;76:453–480. 10.1016/0022-5193(79)90013-4 439915

[52] Singh A, Hespanha JP. Stochastic hybrid systems for studying biochemical processes. Philosophical Transactions of the Royal Society of London A: Mathematical, Physical and Engineering Sciences. 2010;368:4995–5011. 10.1098/rsta.2010.0211 20921008

[53] Daigle BJ, Soltani M, Petzold LR, Singh A. Inferring single-cell gene expression mechanisms using stochastic simulation. Bioinformatics. 2015;31:1428–1435. 10.1093/bioinformatics/btv007 25573914 PMC4492418

[54] Lagershausen S. Performance Analysis of Closed Queueing Networks Lecture Notes in Economics and Mathematical Systems. Springer; 2013.

[55] Buchholz P, Kriege J, Felko I. Input Modeling with Phase-Type Distributions and Markov Models. Springer; 2014.

[56] Hespanha JP, Singh A. Stochastic Models for Chemically Reacting Systems Using Polynomial Stochastic Hybrid Systems. International Journal of Robust and Nonlinear Control. 2005;15:669–689. 10.1002/rnc.1017

[57] Singh A, Hespanha JP. Approximate Moment Dynamics for Chemically Reacting Systems. IEEE Transactions on Automatic Control. 2011;56:414–418. 10.1109/TAC.2010.2088631

[58] Gomez-Uribe CA, Verghese GC. Mass Fluctuation Kinetics: Capturing Stochastic Effects in Systems of Chemical Reactions through Coupled Mean-Variance Computations. Journal of Chemical Physics. 2007;126:024109 10.1063/1.2408422 17228945

[59] Lee CH, Kim K, Kim P. A moment closure method for stochastic reaction networks. Journal of Chemical Physics. 2009;130:134107 10.1063/1.3103264 19355717

[60] Goutsias J. Classical versus stochastic kinetics modeling of biochemical reaction systems. Biophysical Journal. 2007;92:2350–2365. 10.1529/biophysj.106.093781 17218456 PMC1864832

[61] Gillespie CS. Moment-closure approximations for mass-action models. IET systems biology. 2009;3:52–58. 10.1049/iet-syb:20070031 19154084

[62] Soltani M, Vargas-Garcia CA, Singh A. Conditional moment closure schemes for studying stochastic dynamics of genetic circuits. IEEE Transactions on Biomedical Systems and Circuits. 2015;9:518–526. 10.1109/TBCAS.2015.2453158 26336146

[63] Wang H, Yuan Z, Liu P, Zhou T. Division time-based amplifiers for stochastic gene expression. Molecular BioSystems. 2015;11:2417–2428. 10.1039/C5MB00391A 26178011

[64] Hilfinger A, Paulsson J. Separating intrinsic from extrinsic fluctuations in dynamic biological systems. Proceedings of the National Academy of Sciences. 2011;108:12167–12172. 10.1073/pnas.1018832108 PMC3141918 21730172

[65] Singh A, Soltani M. Quantifying Intrinsic and Extrinsic Variability in Stochastic Gene Expression Models. PLOS ONE. 2013;8:e84301 10.1371/journal.pone.0084301 24391934 PMC3877255

[66] Shahrezaei V, Ollivier JF, Swain PS. Colored extrinsic fluctuations and stochastic gene expression. Molecular Systems Biology. 2008;4:196 10.1038/msb.2008.31 18463620 PMC2424296

[67] Scott M, Ingalls B, Kaern M. Estimations of intrinsic and extrinsic noise in models of nonlinear genetic networks. Chaos. 2006;16:026107 10.1063/1.2211787 16822039

[68] Ozbudak EM, Thattai M, Kurtser I, Grossman AD, van Oudenaarden A. Regulation of noise in the expression of a single gene. Nature Genetics. 2002;31:69–73. 10.1038/ng869 11967532

[69] Newman JRS, Ghaemmaghami S, Ihmels J, Breslow DK, Noble M, DeRisi JL, et al Single-cell proteomic analysis of S. cerevisiae reveals the architecture of biological noise. Nature. 2006;441:840–846. 10.1038/nature04785 16699522

[70] Singh A, Razooky B, Cox CD, Simpson ML, Weinberger LS. Transcriptional Bursting from the HIV-1 Promoter Is a Significant Source of Stochastic Noise in HIV-1 Gene Expression. Biophysical Journal. 2010;98:L32–L34. 10.1016/j.bpj.2010.03.001 20409455 PMC2856162

[71] Bar-Even A, Paulsson J, Maheshri N, Carmi M, O’Shea E, Pilpel Y, et al Noise in protein expression scales with natural protein abundance. Nature Genetics. 2006;38:636–643. 10.1038/ng1807 16715097

[72] Yunger S, Rosenfeld L, Garini Y, Shav-Tal Y. Single-allele analysis of transcription kinetics in living mammalian cells. Nature Methods. 2010;7:631–633. 10.1038/nmeth.1482 20639867

[73] Lott SE, Villalta JE, Schroth GP, Luo S, Tonkin LA, Eisen MB. Noncanonical Compensation of Zygotic X Transcription in Early Drosophila melanogaster Development Revealed through Single-Embryo RNA-Seq. PLOS Biology. 2011;9:e1000590 10.1371/journal.pbio.1000590 21346796 PMC3035605

[74] Walker N, Nghe P, Tans SJ. Generation and filtering of gene expression noise by the bacterial cell cycle. BMC Biology. 2016;14:1–10. 10.1186/s12915-016-0231-z 26867568 PMC4750204

[75] Keren L, van Dijk D, Weingarten-Gabbay S, Davidi D, Jona G, Weinberger A, et al Noise in gene expression is coupled to growth rate. Genome Research. 2015;25:1893–1902. 10.1101/gr.191635.115 26355006 PMC4665010

[76] Siegal-Gaskins D, Crosson S. Tightly Regulated and Heritable Division Control in Single Bacterial Cells. Biophysical Journal. 2008;95:2063–2072. 10.1529/biophysj.108.128785 18469083 PMC2483777

[77] Cerulus B, New AM, Pougach K, Verstrepen KJ. Noise and Epigenetic Inheritance of Single-Cell Division Times Influence Population Fitness. Current Biology. 2016; 26: 1138–1147. 10.1016/j.cub.2016.03.010 27068419 PMC5428746

[78] Taheri-Araghi S, Bradde S, Sauls JT, Hill NS, Levin PA, Paulsson J, et al Cell-Size Control and Homeostasis in Bacteria. Current Biology. 2015;25:385–391. 10.1016/j.cub.2014.12.009 25544609 PMC4323405

[79] Adiciptaningrum A, Osella M, Moolman MC, Lagomarsino MC, Tans SJ. Stochasticity and homeostasis in the E. coli replication and division cycle. Scientific Reports. 2015;5:18261 10.1038/srep18261 26671779 PMC4680914

[80] Friedman N, Cai L, Xie XS. Linking stochastic dynamics to population distribution: an analytical framework of gene expression. Physical Review Letters. 2006;97:168302 10.1103/PhysRevLett.97.168302 17155441

[81] Paulsson J, Ehrenberg M. Random Signal Fluctuations Can Reduce Random Fluctuations in Regulated Components of Chemical Regulatory Networks. Physical Review Letters. 2000; 84:5447–5450. 10.1103/PhysRevLett.84.5447 10990965

[82] Sherman MS, Cohen BA. A Computational Framework for Analyzing Stochasticity in Gene Expression. PLOS Computational Biology. 2014;10:e1003596 10.1371/journal.pcbi.1003596 24811315 PMC4014403

[83] Golding I, Paulsson J, Zawilski SM, Cox EC. Real-time kinetics of gene activity in individual bacteria. Cell. 2005;123:1025–1036. 10.1016/j.cell.2005.09.031 16360033

[84] McAdams HH, Arkin A. Stochastic mechanisms in gene expression. Proceedings of the National Academy of Sciences. 1997; 94:814–819. 9023339 10.1073/pnas.94.3.814 PMC19596

[85] Cai L, Friedman N, Xie XS. Stochastic protein expression in individual cells at the single molecule level. Nature. 2006;440:358–362. 10.1038/nature04599 16541077

[86] Kumar N, Singh A, Kulkarni RV. Transcriptional bursting in gene expression: analytical results for general stochastic models. PLOS Computational Biology. 2015;11:e1004292 10.1371/journal.pcbi.1004292 26474290 PMC4608583

[87] Singh A. Transient Changes in Intercellular Protein Variability Identify Sources of Noise in Gene Expression. Biophysical Journal. 2014;107:2214–2220. 10.1016/j.bpj.2014.09.017 25418106 PMC4223191

[88] Osella M, Nugent E, Lagomarsino MC. Concerted control of *Escherichia coli* cell division. Proceedings of the National Academy of Sciences. 2014;111:3431–3435. 10.1073/pnas.1313715111 PMC3948223 24550446

[89] Robert L, Hoffmann M, Krell N, Aymerich S, Robert J, Doumic M. Division in *Escherichia coli* is triggered by a size-sensing rather than a timing mechanism. BMC Biology. 2014;12:17 10.1186/1741-7007-12-17 24580833 PMC4016582

[90] Kafri R, Levy J, Ginzberg MB, Oh S, Lahav G, Kirschner MW. Dynamics extracted from fixed cells reveal feedback linking cell growth to cell cycle. Nature. 2013;494:480–483. 10.1038/nature11897 23446419 PMC3730528

[91] Ghusinga KR, Vargas-Garcia CA, Singh A. A mechanistic stochastic framework for regulating bacterial cell division. Scientific Reports. 2016; 6: 30229 10.1038/srep30229 27456660 PMC4960620

[92] Amir A. Cell Size Regulation in Bacteria. Physical Review Letters. 2014;112:208102 10.1103/PhysRevLett.112.208102

[93] Campos M, Surovtsev IV, Kato S, Paintdakhi A, Beltran B, Ebmeier SE, et al A Constant Size Extension Drives Bacterial Cell Size Homeostasis. Cell. 2014;159:1433–1446. 10.1016/j.cell.2014.11.022 25480302 PMC4258233

[94] Padovan-Merhar O, Nair GP, Biaesch AG, Mayer A, Scarfone S, Foley SW, et al Single Mammalian Cells Compensate for Differences in Cellular Volume and DNA Copy Number through Independent Global Transcriptiona Mechanisms. Molecular Cell. 2015;58:339–352. 10.1016/j.molcel.2015.03.005 25866248 PMC4402149

[95] Suter DM, Molina N, Gatfield D, Schneider K, Schibler U, Naef F. Mammalian genes are transcribed with widely different bursting kinetics. Science. 2011;332:472–474. 10.1126/science.1198817 21415320

[96] Brown CR, Mao C, Falkovskaia E, Jurica MS, Boeger H. Linking Stochastic Fluctuations in Chromatin Structure and Gene Expression. PLOS Biology. 2013;11:e1001621 10.1371/journal.pbio.1001621 23940458 PMC3735467

[97] Raj A, Peskin CS, Tranchina D, Vargas DY, Tyagi S. Stochastic mRNA synthesis in mammalian cells. PLOS Biology. 2006;4:e309 10.1371/journal.pbio.0040309 17048983 PMC1563489

[98] Hornung G, Bar-Ziv R, Rosin D, Tokuriki N, Tawfik DS, Oren M, et al Noise-mean relationship in mutated promoters. Genome Research. 2012;22:2409–2417. 10.1101/gr.139378.112 22820945 PMC3514670

[99] Singh A, Razooky BS, Dar RD, Weinberger LS. Dynamics of protein noise can distinguish between alternate sources of gene-expression variability. Molecular Systems Biology. 2012;8:607 10.1038/msb.2012.38 22929617 PMC3435505

[100] Dar RD, Razooky BS, Singh A, Trimeloni T, McCollum J, Cox C, et al Transcriptional burst frequency and burst size are equally modulated across the human genome. Proceedings of the National Academy of Sciences. 2012;109:17454–17459. 10.1073/pnas.1213530109 PMC3491463 23064634

[101] Corrigan AM, Chubb JR. Regulation of Transcriptional Bursting by a Naturally Oscillating Signal. Current Biology. 2014;24:205–211. 10.1016/j.cub.2013.12.011 24388853 PMC3928820

[102] Bothma JP, Garcia HG, Esposito E, Schlissel G, Gregor T, Levine M. Dynamic regulation of eve stripe 2 expression reveals transcriptional bursts in living Drosophila embryos. Proceedings of the National Academy of Sciences. 2014;111:10598–10603. 10.1073/pnas.1410022111 PMC4115566 24994903

[103] Chubb JR, Trcek T, Shenoy SM, Singer RH. Transcriptional Pulsing of a Developmental Gene. Current Biology. 2006;16:1018–1025. 10.1016/j.cub.2006.03.092 16713960 PMC4764056

[104] Chong S, Chen C, Ge H, Xie XS. Mechanism of Transcriptional Bursting in Bacteria. Cell. 2014;158:314–326. 10.1016/j.cell.2014.05.038 25036631 PMC4105854

