# Supplementary material for: Intercellular Variability in Protein Levels from Stochastic Expression and Noisy Cell Cycle Processes
Source: PLoS Comput Biol. 2016 Aug 18;12(8):e1004972. doi: 10.1371/journal.pcbi.1004972 (PMC4990281; doi:10.1371/journal.pcbi.1004972)
Supplement: S1 Text — Additional proofs and simulations. (PDF) [file pcbi.1004972.s001.pdf]

Supplementary Material

Intercellular variability in protein levels from stochastic expression and noisy cell cycle processes

Contents

A Mean of protein in the presence of cell cycle variations 2

B Moment dynamics of hybrid model introduced in Figure 2B 6

C Moment dynamics of hybrid model introduced in Figure 2C 9

D Effect of mean protein level on partitioning noise 12

D.1 Partitioning noise for constant variance 12

E Second and third-order moment dynamics of the full model 14

F Contribution of different sources of stochasticity in protein by taking into account gene duplication 16

F.1 Mean of protein count level in the presence of gene duplication 18

F.2 Noise in protein count level contributed from cell cycle time variations . . . 22

F.3 Noise in protein count level contributed from random partitioning 26

F.4 Noise in protein count level contributed from stochastic production 29

F.5 Effect of gene duplication time on intrinsic noise 31

G Noise level in unstable protein 33

H Simulation results 36

Appendix A

Mean of protein in the presence of cell cycle variations

Based on standard stochastic formulation of chemical kinetics [1, 2], the model introduced in Figure 2A coupled with phase-type distribution introduced in Figure 3 contains the following stochastic events

Event	Reset	Propensity
Protein production	$x(t) \mapsto x(t)+u$	$k_x p''_u$
Phase-type Evolution	$g_{ij}(t) \mapsto g_{ij}(t)-1,$ $g_{i(j+1)}(t) \mapsto g_{i(j+1)}(t)+1$	$ikg_{ij},$ $i \in \{2,\dots,n\},$ $j \in \{1,\dots,i-1\}$
Cell-division	$x(t_s) \mapsto x_+(t_s),$ $g_{jj}(t_s) \mapsto 0,$ $g_{i1}(t_s) \mapsto g_{i1}(t_s)+1$	$kp_i \sum_{j=1}^{n}(jg_{jj}),$ $i \in \{1,\dots,n\}$

Note that $x_+(t_s)$ is protein level after division, characteristics of $x_+(t_s)$ is related to protein level before division as shown in equation (5) of the main text. Whenever an event occurs, protein level and states of phase-type distribution change based on the stoichiometries shown in the second column of the table. The third column of table shows event propensity function $\psi(x, g_{ij})$, which determines how often reactions occur, i.e., the probability that an event occurs in the next infinitesimal time interval $(t, t+dt]$ is $\psi(x, g_{ij})dt$. Protein production is a stochastic event which happens in bursts, each burst generates B molecules where B is a general random variable with distribution

$$\text{Probability}\{B = u\} = p''_u, \quad u \in \{0, 1, \dots, \infty\}. \tag{A.1}$$

The probability of having a burst in the time interval $(t, t+dt]$ is $k_x p''_u dt$. Events related to time evolution of phase-type distribution happen with a constant rate ik.

Cell division changes both the level of protein and states of phase-type. This event contains start of new cell cycle, hence whenever this event occurs, the last state of phase-type distribution resets to zero, and a new cell cycle which is sum of i exponentials starts with probability p_i; protein count level also resets to $x_+(t_s)$. The probability of cell division and starting a new cell cycle from state g_{i1} in the time interval $(t, t+dt]$ is $kp_i \sum_{j=1}^{n}(jg_{jj})dt$.

Theorem 1 of [3] gives the time derivative of the expected value of any function $\varphi(x, g_{ij})$ as

$$\frac{d\langle\varphi(x, g_{ij})\rangle}{dt} = \left\langle \sum_{Events} \Delta\varphi(x, g_{ij}) \times \psi(x, g_{ij}) \right\rangle, \tag{A.2}$$

where $\Delta\varphi(x, g_{ij})$ is a change in φ when an event occurs. Based on this setup, mean dynamics of protein can be written by choosing φ to be x

$$\begin{aligned} \frac{d\langle x\rangle}{dt} &= k_x\langle B\rangle + k\sum_{j=1}^{n} j\left(\left\langle (\frac{x}{2} - x)g_{jj}\right\rangle\right) \Rightarrow \\ \frac{d\langle x\rangle}{dt} &= k_x\langle B\rangle - \frac{k}{2}\sum_{j=1}^{n}\left(j\langle xg_{jj}\rangle\right), \end{aligned} \tag{A.3}$$

where we replaced conditional expected value of x_+ by $x/2$ based on relation between statistical properties of x_+ and x shown in equation (5).

Dynamics of $\langle x\rangle$ is not closed and depends to moments $\langle xg_{jj}\rangle$, hence in order to have a closed set of equations we add new moments dynamics by selecting φ to be xg_{ij}. We do it in two steps: first we write the moment dynamics of $\langle xg_{11}\rangle$

$$\frac{d\langle xg_{11}\rangle}{dt} = k_x\langle B\rangle\langle g_{11}\rangle + \frac{k}{2}p_1\left\langle xg_{11}^2\right\rangle - kp_1\left\langle xg_{11}^2\right\rangle - k\sum_{i=2}^{n} p_i\left\langle xg_{11}\right\rangle. \tag{A.4}$$

In the equation (9) of the main text it has been shown that

$$\langle g_{ij}^n x^m \rangle = \langle g_{ij} x^m \rangle, \quad n \in \{1, 2, \dots\}, \tag{A.5}$$

thus the term $\langle x g_{11}^2 \rangle$ will simplify as

$$\left\langle x g_{11}^2 \right\rangle = \langle x g_{11} \rangle \,, \tag{A.6}$$

and the dynamics of $\langle x g_{11} \rangle$ can be written as

$$\frac{d\langle x g_{11} \rangle}{dt} = k_x \langle B \rangle \langle g_{11} \rangle + \frac{k}{2} p_1 \langle x g_{11} \rangle - k \langle x g_{11} \rangle \,. \tag{A.7}$$

In the second step we write dynamics of the moments of the form $\langle x g_{ij} \rangle$ other than $\langle x g_{11} \rangle$

$$\frac{d\langle x g_{i1} \rangle}{dt} = k_x \langle B \rangle \langle g_{i1} \rangle + k p_i \sum_{j=1}^{n} \left(j \left\langle (\frac{x}{2} + \frac{x}{2} g_{i1} - x g_{i1}) g_{jj} \right\rangle \right) - ik \langle x g_{i1} \rangle, \tag{A.8a}$$

$$\frac{d\langle x g_{ij} \rangle}{dt} = k_x \langle B \rangle \langle g_{ij} \rangle - ik \langle x g_{ij} \rangle + ik \langle x g_{i(j-1)} \rangle, \quad j \in \{2, \dots, i\}, \tag{A.8b}$$

where dynamics of $\langle x g_{i1} \rangle$ can be written as

$$\frac{d\langle x g_{i1} \rangle}{dt} = k_x \langle B \rangle \langle g_{i1} \rangle + k p_i \sum_{j=1}^{n} \left(j \left\langle \frac{x}{2} g_{jj} \right\rangle \right) + k p_i \sum_{j=1}^{n} \left(-j \left\langle \frac{x}{2} g_{i1} g_{jj} \right\rangle \right) - ik \langle x g_{i1} \rangle. \tag{A.9}$$

The equation (10) in the main text shows that

$$\langle g_{ij} g_{rq} x^m \rangle = 0, \text{ if } i \neq r \text{ or } j \neq q, \tag{A.10}$$

hence $\sum_{j=1}^{n}\left(j\left\langle \frac{x}{2}g_{i1}g_{jj}\right\rangle\right)=0$, and equation (A.9) simplifies to

$$\frac{d\langle xg_{i1}\rangle}{dt}=k_x\langle B\rangle\langle g_{i1}\rangle+\frac{k}{2}p_i\sum_{j=1}^{n}\left(j\left\langle xg_{jj}\right\rangle\right)-ik\langle xg_{i1}\rangle. \tag{A.11}$$

Further based on Figure 3 in the main text the probability of selecting a branch of i exponentials is p_i, and because all the transitions happen with a constant rate ik, hence mean of each of these i states is

$$\overline{\langle g_{ij}\rangle}=\frac{p_i}{i}. \tag{A.12}$$

This also can be seen by solving moment dynamics of $\langle g_{ij}\rangle$

$$\frac{d\langle g_{i1}\rangle}{dt}=kp_i\sum_{j=1}^{n}\left(j\langle g_{jj}\rangle\right)-ik\langle g_{i1}\rangle, \tag{A.13a}$$

$$\frac{d\langle g_{ij}\rangle}{dt}=ik\langle g_{i(j-1)}\rangle-ik\langle g_{ij}\rangle,\ j=\{2,\dots,i\}. \tag{A.13b}$$

Overall equations (A.7), (A.8b), (A.13), and (A.11) can be compactly written as shown in equations (11)-(14).

Appendix B

Moment dynamics of hybrid model introduced in Figure 2B

Stochastic hybrid system introduced in Figure 2B coupled with phase-type distribution contains the following stochastic events

Event	Reset	Propensity
Phase-type Evolution	$g_{ij}(t) \mapsto g_{ij}(t)-1,$ $g_{i(j+1)}(t) \mapsto g_{i(j+1)}(t)+1$	$ikg_{ij},$ $i \in \{2,\dots,n\},$ $j \in \{1,\dots,i-1\}$
Cell-division	$x(t_s) \mapsto x(t_s)/2,$ $g_{jj}(t_s) \mapsto 0,$ $g_{i1}(t_s) \mapsto g_{i1}(t_s)+1$	$kp_i \sum_{j=1}^{n}(jg_{jj}),$ $i \in \{1,\dots,n\}$

and deterministic protein production dynamics

$$\dot{x} = k_x \langle B \rangle. \tag{B.1}$$

Time derivative of the expected value of any function $\varphi(x, g_{ij})$ for this hybrid system can be written as [3]

$$\frac{d\langle \varphi(x, g_{ij}) \rangle}{dt} = \left\langle \sum_{Events} \Delta\varphi(x, g_{ij}) \times \psi(x, g_{ij}) \right\rangle + \left\langle \frac{\partial \varphi(x, g_{ij})}{\partial x} k_x \langle B \rangle \right\rangle, \tag{B.2}$$

where the first term in the right-hand side is contributed from stochastic events and the second term is contributed from deterministic protein production dynamics. Based on

this equation, the mean dynamics of the protein is calculated by choosing φ to be x

$$\frac{d\langle x\rangle}{dt} = k_x\langle B\rangle - \frac{k}{2}\sum_{j=1}^{n}\left(j\left\langle xg_{jj}\right\rangle\right), \tag{B.3}$$

which is the same as equation (A.3). In addition to mean, dynamics of $\langle xg_{ij}\rangle$ are also equal to their equation in the previous section.

The second order moment dynamics of protein can be expressed by choosing φ to be x^2

$$\frac{d\langle x^2\rangle}{dt} = 2k_x\langle B\rangle\langle x\rangle + k\sum_{j=1}^{n}\left(j\left\langle\left(\left(\frac{x}{2}\right)^2 - x^2\right)g_{jj}\right\rangle\right), \tag{B.4}$$

which can be simplified as

$$\frac{d\langle x^2\rangle}{dt} = 2k_x\langle B\rangle\langle x\rangle - \frac{3k}{4}\sum_{j=1}^{n}\left(j\left\langle x^2 g_{jj}\right\rangle\right). \tag{B.5}$$

In order to have a closed set of equations we select φ to be of the form $x^2 g_{ij}$. At the first step we write moment dynamics of $\langle x^2 g_{11}\rangle$

$$\frac{d\langle x^2 g_{11}\rangle}{dt} = 2k_x\langle B\rangle\langle xg_{11}\rangle + \frac{k}{4}p_1\left\langle x^2 g_{11}^2\right\rangle - kp_1\left\langle x^2 g_{11}^2\right\rangle - k\sum_{i=2}^{n}p_i\langle x^2 g_{11}\rangle. \tag{B.6}$$

Based on equation (9) of the main text, the term $\langle x^2 g_{11}^2\rangle$ simplifies as

$$\left\langle x^2 g_{11}^2\right\rangle = \left\langle x^2 g_{11}\right\rangle, \tag{B.7}$$

hence dynamics of $\langle x^2 g_{11}\rangle$ will be

$$\frac{d\langle x^2 g_{11}\rangle}{dt} = 2k_x\langle B\rangle\langle xg_{11}\rangle + \frac{k}{4}p_1\left\langle x^2 g_{11}\right\rangle - k\langle x^2 g_{11}\rangle. \tag{B.8}$$

In the second step, we write dynamics of moments $\langle x^2 g_{ij} \rangle$ when $g_{ij} \neq g_{11}$

$$\frac{d\langle x^2 g_{i1} \rangle}{dt} = 2k_x \langle B \rangle \langle x g_{i1} \rangle + k p_i \sum_{j=1}^{n} \left(j \left\langle \left(\frac{x^2}{4} + \frac{x^2}{4} g_{i1} - x^2 g_{i1} \right) g_{jj} \right\rangle \right) - ik \langle x^2 g_{i1} \rangle, \tag{B.9a}$$

$$\frac{d\langle x^2 g_{ij} \rangle}{dt} = 2k_x \langle B \rangle \langle x g_{ij} \rangle - ik \langle x^2 g_{ij} \rangle + ik \langle x^2 g_{(i-1)j} \rangle, \; j = \{2, \ldots, i\}, \tag{B.9b}$$

where dynamics of $\langle x^2 g_{i1} \rangle$ can be shown to follow

$$\frac{d\langle x^2 g_{i1} \rangle}{dt} = 2k_x \langle B \rangle \langle x g_{i1} \rangle + \frac{k}{4} p_i \sum_{j=1}^{n} \left(j \left\langle x^2 g_{jj} \right\rangle \right) - \frac{3k}{4} p_i \sum_{j=1}^{n} \left(j \left\langle x^2 g_{i1} g_{jj} \right\rangle \right) - ik \langle x^2 g_{i1} \rangle. \tag{B.10}$$

Based on equation (10) in the main text $\sum_{j=1}^{n} \left(j \left\langle x^2 g_{i1} g_{jj} \right\rangle \right) = 0$, thus equation (B.10) simplifies to

$$\frac{d\langle x^2 g_{i1} \rangle}{dt} = 2k_x \langle B \rangle \langle x g_{i1} \rangle + \frac{k}{4} p_i \sum_{j=1}^{n} \left(j \left\langle x^2 g_{jj} \right\rangle \right) - ik \langle x^2 g_{i1} \rangle. \tag{B.11}$$

Equations (B.8), (B.9b), and (B.11) can be compactly written as equations (19) and (20) in the main text.

Appendix C

Moment dynamics of hybrid model introduced in Figure 2C

Stochastic hybrid system introduced in Figure 2C coupled with phase-type distribution contains the following stochastic events

Event	Reset	Propensity
Phase-type Evolution	$g_{ij}(t) \mapsto g_{ij}(t)-1$, $g_{i(j+1)}(t) \mapsto g_{i(j+1)}(t)+1$	ikg_{ij}, $i \in \{2,\ldots,n\}$, $j \in \{1,\ldots,i-1\}$
Cell-division	$x(t_s) \mapsto x_+(t_s)$, $g_{jj}(t_s) \mapsto 0$, $g_{i1}(t_s) \mapsto g_{i1}(t_s)+1$	$kp_i \sum_{j=1}^{n}(jg_{jj})$, $i \in \{1,\ldots,n\}$

and deterministic protein production dynamics

$$\dot{x} = k_x \langle B \rangle. \tag{C.1}$$

Note that in this model $x(t)$ is a continuous random variable, thus we also use a continuous distribution to describe $x_+(t_s)$, however statistical properties of $x_+(t_s)$ is still given by (5). For this model we still can use equation (B.2) to derive moment dynamics; equations describing time evolution of mean and $\langle x g_{ij} \rangle$ are the same as previous models, thus mean of protein for this model is equal to its value in Appendix A. The second order moment

dynamics of protein can be written by choosing φ to be x^2 in equation (B.2)

$$\frac{d\langle x^2\rangle}{dt} = 2k_x\langle B\rangle\langle x\rangle + k\sum_{j=1}^{n}\left(j\left\langle\left(\frac{x^2}{4}+\frac{\alpha x}{4}-x^2\right)g_{jj}\right\rangle\right), \tag{C.2}$$

where conditional expected value of x_+^2 is substituted based on equation (5). Dynamics of $\langle x^2\rangle$ can be simplified as

$$\frac{d\langle x^2\rangle}{dt} = 2k_x\langle B\rangle\langle x\rangle + \frac{\alpha k}{4}\sum_{j=1}^{n}\left(j\left\langle xg_{jj}\right\rangle\right) - \frac{3k}{4}\sum_{j=1}^{n}\left(j\left\langle x^2 g_{jj}\right\rangle\right). \tag{C.3}$$

The same as before we add dynamics of the form $\langle x^2 g_{ij}\rangle$ to have a closed set of dynamics. First we add dynamics of $\langle x^2 g_{11}\rangle$

$$\frac{d\langle x^2 g_{11}\rangle}{dt} = 2k_x\langle B\rangle\langle xg_{11}\rangle + \frac{\alpha k}{4}p_1\left\langle xg_{11}^2\right\rangle + \frac{k}{4}p_1\left\langle x^2 g_{11}^2\right\rangle - kp_1\left\langle x^2 g_{11}^2\right\rangle - k\sum_{i=2}^{n}p_i\langle x^2 g_{11}\rangle, \tag{C.4}$$

Based on equation (9) of the main text dynamics of $\langle x^2 g_{11}\rangle$ simplifies to

$$\frac{d\langle x^2 g_{11}\rangle}{dt} = 2k_x\langle B\rangle\langle xg_{11}\rangle + \frac{\alpha k}{4}p_1\left\langle xg_{11}\right\rangle + \frac{k}{4}p_1\left\langle x^2 g_{11}\right\rangle - k\langle x^2 g_{11}\rangle. \tag{C.5}$$

Now we express dynamics of moments $\langle x^2 g_{ij}\rangle$ for $g_{ij}\neq g_{11}$

$$\frac{d\langle x^2 g_{i1}\rangle}{dt} = 2k_x\langle B\rangle\langle xg_{i1}\rangle + kp_i\sum_{j=1}^{n}\left(j\left\langle\left(\frac{x^2}{4}+\frac{x^2}{4}g_{i1}+\frac{\alpha x}{4}+\frac{\alpha x}{4}g_{i1}-x^2 g_{i1}\right)g_{jj}\right\rangle\right) - ik\langle x^2 g_{i1}\rangle, \tag{C.6a}$$

$$\frac{d\langle x^2 g_{ij}\rangle}{dt} = 2k_x\langle B\rangle\langle xg_{ij}\rangle - ik\langle x^2 g_{ij}\rangle + ik\langle x^2 g_{(i-1)j}\rangle,\ j=\{2,\ldots,i\}, \tag{C.6b}$$

where dynamics of $\langle x^2 g_{i1}\rangle$ can be shown as

$$\begin{aligned}\frac{d\langle x^2 g_{i1}\rangle}{dt} =& 2k_x\langle B\rangle\langle x g_{i1}\rangle + \frac{\alpha k}{4} p_i \sum_{j=1}^{n} \left(j\left\langle x g_{jj}\right\rangle\right) + \frac{k}{4} p_i \sum_{j=1}^{n} \left(j\left\langle x^2 g_{jj}\right\rangle\right) \\ &+ \frac{\alpha k}{4} p_i \sum_{j=1}^{n} \left(j\left\langle x g_{i1} g_{jj}\right\rangle\right) - \frac{3k}{4} p_i \sum_{j=1}^{n} \left(j\left\langle x^2 g_{i1} g_{jj}\right\rangle\right) - ik\langle x^2 g_{i1}\rangle.\end{aligned} \tag{C.7}$$

Based on equation (10) in the main text $\sum_{j=1}^{n}\left(j\left\langle x^2 g_{i1} g_{jj}\right\rangle\right) = 0$, and $\sum_{j=1}^{n}\left(j\left\langle x g_{i1} g_{jj}\right\rangle\right) = 0$, hence equation (C.7) simplifies to

$$\frac{d\langle x^2 g_{i1}\rangle}{dt} = 2k_x\langle B\rangle\langle x g_{i1}\rangle + \frac{\alpha k}{4} p_i \sum_{j=1}^{n} \left(j\left\langle x g_{jj}\right\rangle\right) + \frac{k}{4} p_i \sum_{j=1}^{n} \left(j\left\langle x^2 g_{jj}\right\rangle\right) - ik\langle x^2 g_{i1}\rangle. \tag{C.8}$$

Equations (C.3), (C.5), (C.6b), and (C.8) can be compactly written as equations (28)-(30) in the main text.

Appendix D

Effect of mean protein level on partitioning noise

Right before division, the phase type is in one of its last states. Hence the mean number of proteins before division, can be derived by conditioning on the phase type being on the last stages G_{ii}

$$\overline{\langle x \mid g_{ii} = 1 \rangle} = \frac{\overline{\langle x g_{ii} \rangle}}{\overline{\langle g_{ii} \rangle}}, \quad i \in \{1, 2, \ldots, n\}. \tag{D.1}$$

Using (D.1) and moment $\langle x g_{ii} \rangle$ obtained from (16) in the main article, we derive the mean number of proteins right before

$$\overline{\langle x | g_{ii} = 1 \rangle} = 2k_x \langle B \rangle \langle T \rangle, \quad i \in \{1, 2, \ldots, n\}. \tag{D.2}$$

D.1 Partitioning noise for constant variance

Suppose that the noise added by partitioning is independent of the protein level before division, i.e.,

$$\langle x_+(t_s) | x(t_s) \rangle = \frac{x(t_s)}{2}, \quad \left\langle x_+^2(t_s) - \langle x_+(t_s) \rangle^2 \middle| x(t_s) \right\rangle = \alpha, \tag{D.3}$$

Then the time evolution of $\langle x^2 \rangle$ and $\langle x^2 g_{ij} \rangle$ changes to

$$\frac{d\langle x^2\rangle}{dt} = 2k_x\langle B\rangle\langle x\rangle + \alpha k\sum_{j=1}^{n}\left(j\langle g_{jj}\rangle\right) - \frac{3}{4}k\sum_{j=1}^{n}\left(j\langle x^2 g_{jj}\rangle\right), \tag{D.4a}$$

$$\frac{d\langle x^2 g_{i1}\rangle}{dt} = 2k_x\langle B\rangle\langle x g_{i1}\rangle + \frac{k}{4}p_i\sum_{j=1}^{n}\left(j\langle x^2 g_{jj}\rangle\right) + \alpha k p_i\sum_{j=1}^{n}\left(j\langle g_{jj}\rangle\right) - ik\langle x^2 g_{i1}\rangle, \tag{D.4b}$$

$$\frac{d\langle x^2 g_{ij}\rangle}{dt} = 2k_x\langle B\rangle\langle x g_{ij}\rangle - ik\langle x^2 g_{ij}\rangle + ik\langle x^2 g_{(i-1)j}\rangle,\ j = \{2,\ldots,i\}. \tag{D.4c}$$

Taking a similar approach as we used in the main article results in

$$\overline{\langle x^2\rangle} = k_x^2\langle B\rangle^2\frac{\langle T^3\rangle + 4CV_T^2\langle T\rangle^3 + 6\langle T\rangle^3}{3\langle T\rangle} + \frac{4\alpha}{3}. \tag{D.5}$$

Finding CV^2 of the protein level and subtracting the extrinsic noise found in (24) results in

$$CV_R^2 = \frac{4\alpha}{3}\frac{1}{\overline{\langle x\rangle}^2}. \tag{D.6}$$

Appendix E

Second and third-order moment dynamics of the full model

Based on model introduced in Appendix A, second order moment dynamics of protein is expressed by choosing φ to be x^2 in equation (A.2),

$$\frac{d\langle x^2\rangle}{dt} = k_x\langle B^2\rangle + 2k_x\langle B\rangle\langle x\rangle + k\sum_{j=1}^{n}\left(j\left\langle\left(\frac{x^2}{4}+\frac{\alpha x}{4}-x^2\right)g_{jj}\right\rangle\right), \tag{E.1}$$

where conditional expected value of x_+^2 is substituted based on equation (5). Dynamics of $\langle x^2\rangle$ can be simplified as

$$\frac{d\langle x^2\rangle}{dt} = k_x\langle B^2\rangle + 2k_x\langle B\rangle\langle x\rangle + \frac{\alpha k}{4}\sum_{j=1}^{n}\left(j\left\langle x g_{jj}\right\rangle\right) - \frac{3k}{4}\sum_{j=1}^{n}\left(j\left\langle x^2 g_{jj}\right\rangle\right). \tag{E.2}$$

The same as before we add dynamics of the form $\langle x^2 g_{ij}\rangle$ to have a closed set of moments. First we write dynamics of $\langle x^2 g_{11}\rangle$

$$\frac{d\langle x^2 g_{11}\rangle}{dt} = k_x\langle B^2\rangle\langle g_{11}\rangle + 2k_x\langle B\rangle\langle x g_{11}\rangle + \frac{\alpha k}{4}p_1\left\langle x g_{11}^2\right\rangle + \frac{k}{4}p_1\left\langle x^2 g_{11}^2\right\rangle - kp_1\left\langle x^2 g_{11}^2\right\rangle - k\sum_{i=2}^{n}p_i\langle x^2 g_{11}\rangle, \tag{E.3}$$

Based on equation (9) of the main text dynamics of $\langle x^2 g_{11}\rangle$ simplifies to

$$\frac{d\langle x^2 g_{11}\rangle}{dt} = k_x\langle B^2\rangle\langle g_{11}\rangle + 2k_x\langle B\rangle\langle x g_{11}\rangle + \frac{\alpha k}{4}p_1\langle x g_{11}\rangle + \frac{k}{4}p_1\left\langle x^2 g_{11}\right\rangle - k\langle x^2 g_{11}\rangle. \tag{E.4}$$

Next, dynamics of moments $\langle x^2 g_{ij}\rangle$ when $g_{ij} \neq g_{11}$ can be written as

$$\begin{aligned}\frac{d\langle x^2 g_{i1}\rangle}{dt} =& k_x\langle B^2\rangle\langle g_{i1}\rangle + 2k_x\langle B\rangle\langle x g_{i1}\rangle \\ &+ kp_i \sum_{j=1}^{n}\left(j\left\langle\left(\frac{x^2}{4}+\frac{x^2}{4}g_{i1}+\frac{\alpha x}{4}+\frac{\alpha x}{4}g_{i1}-x^2 g_{i1}\right)g_{jj}\right\rangle\right) - ik\langle x^2 g_{i1}\rangle,\end{aligned} \tag{E.5a}$$

$$\frac{d\langle x^2 g_{ij}\rangle}{dt} = k_x\langle B^2\rangle\langle g_{ij}\rangle + 2k_x\langle B\rangle\langle x g_{ij}\rangle - ik\langle x^2 g_{ij}\rangle + ik\langle x^2 g_{(i-1)j}\rangle,\ j = \{2,\ldots,i\}, \tag{E.5b}$$

where dynamics of $\langle x^2 g_{i1}\rangle$ can be shown as

$$\begin{aligned}\frac{d\langle x^2 g_{i1}\rangle}{dt} =& k_x\langle B^2\rangle\langle g_{i1}\rangle + 2k_x\langle B\rangle\langle x g_{i1}\rangle + \frac{\alpha k}{4}p_i\sum_{j=1}^{n}\left(j\left\langle x g_{jj}\right\rangle\right) + \frac{k}{4}p_i\sum_{j=1}^{n}\left(j\left\langle x^2 g_{jj}\right\rangle\right) \\ &+ \frac{\alpha k}{4}p_i\sum_{j=1}^{n}\left(j\left\langle x g_{i1} g_{jj}\right\rangle\right) - \frac{3k}{4}p_i\sum_{j=1}^{n}\left(j\left\langle x^2 g_{i1} g_{jj}\right\rangle\right) - ik\langle x^2 g_{i1}\rangle.\end{aligned} \tag{E.6}$$

Based on equation (10) in the main text $\sum_{j=1}^{n}\left(j\left\langle x^2 g_{i1} g_{jj}\right\rangle\right) = 0$ and $\sum_{j=1}^{n}\left(j\left\langle x g_{i1} g_{jj}\right\rangle\right) = 0$, hence equation (E.6) simplifies to

$$\frac{d\langle x^2 g_{i1}\rangle}{dt} = k_x\langle B^2\rangle\langle g_{i1}\rangle + 2k_x\langle B\rangle\langle x g_{i1}\rangle + \frac{\alpha k}{4}p_i\sum_{j=1}^{n}\left(j\left\langle x g_{jj}\right\rangle\right) + \frac{k}{4}p_i\sum_{j=1}^{n}\left(j\left\langle x^2 g_{jj}\right\rangle\right) - ik\langle x^2 g_{i1}\rangle. \tag{E.7}$$

Equations (E.2), (E.4), (E.5b), and (E.7) can be compactly written as equations (35)-(37) in the main text.

Appendix F

Contribution of different sources of stochasticity in protein by taking into account gene duplication

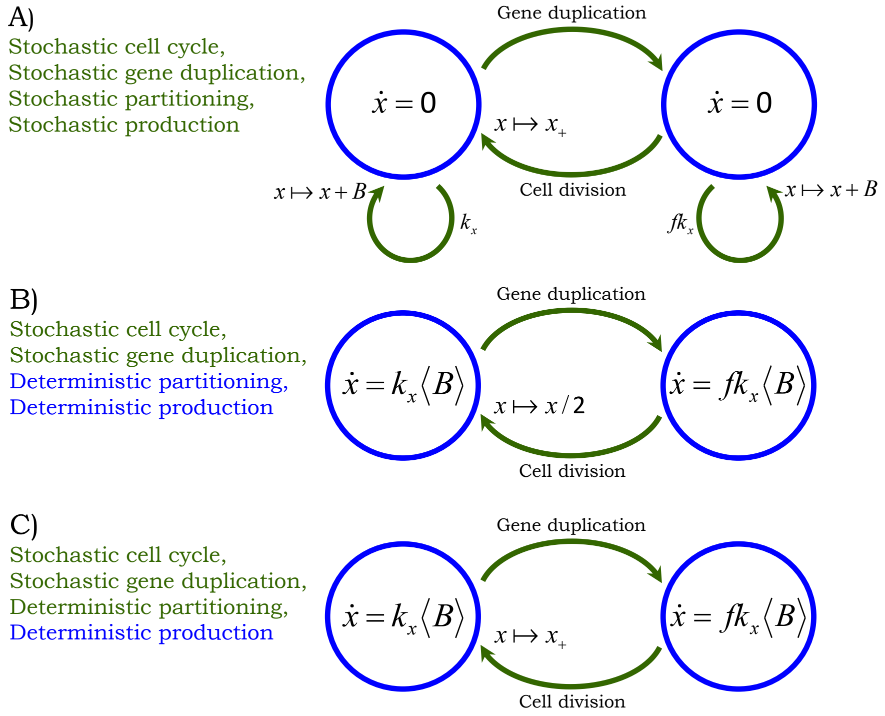

Figure A: **Stochastic hybrid models for quantifying different sources of noise, where gene duplication and cell division times are random events**. **A)** Protein production happens in random bursts with burst frequency k_x. After gene duplication event, burst frequency increases to fk_x ($f > 1$). At the time of division, proteins are randomly distributed between daughter cells, and the protein burst frequency is k_x again. **B)** Protein production is deterministic, and after gene duplication, dynamics of protein production is multiplied by a factor f, i.e., $\dot{x} = fk_x\langle B\rangle$. At the division event, proteins are distributed between daughter cells equally. Thus the only stochastic events are duplication and division events. **C)** Protein production is deterministic, and protein levels are distributed randomly among daughter and mother cells. Thus duplication, division, and partitioning are random events.

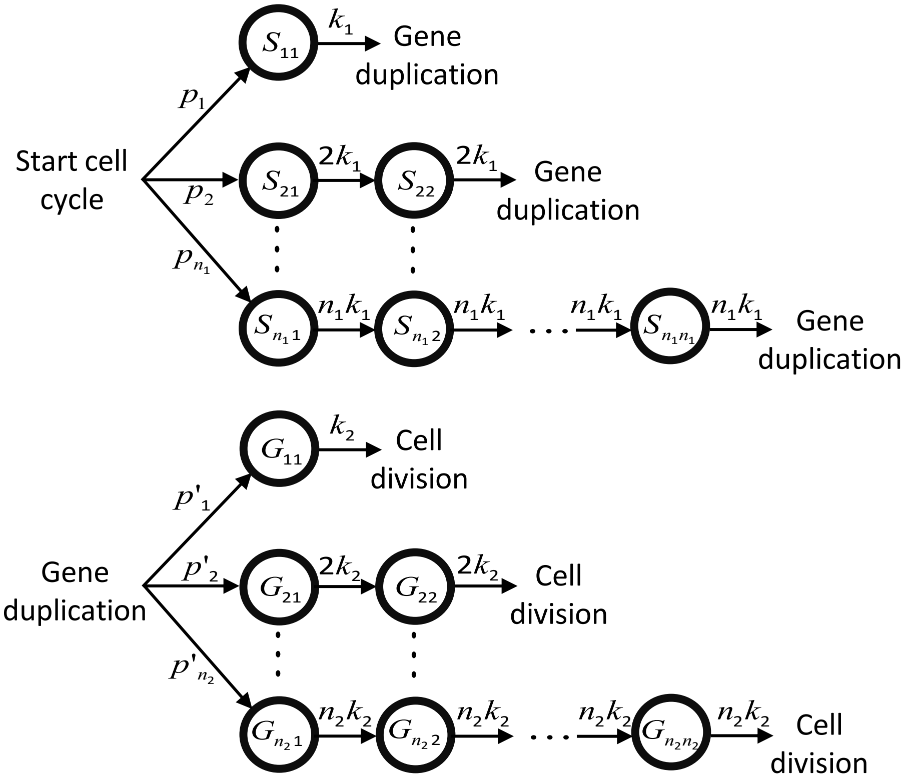

Figure B: **Cell cycle time consists of two time intervals: at the end of the first interval gene duplicates, and at the end of the second one cell divides.** Two independent phase-type distributions are used to model cell cycle time in the presence of genome duplication. The states of the first distribution are denoted by $S_{ij},\ i = \{1, \ldots, n_1\},\ j = \{1, \ldots, i\}$; transition between these states happens at rate ik_1. The states of the second distribution are shown by $G_{ij},\ i = \{1, \ldots, n_2\},\ j = \{1, \ldots, i\}$, and transition between these states occurs at rate ik_2.

We study the contribution of different sources of stochasticity by using models introduced in Figure A. Note that the model in Figure A.A contains both intrinsic and extrinsic sources of randomness. Model in Figure A.B just include extrinsic noise, and finally model in Figure A.C contains extrinsic noise and one source of intrinsic noise (partitioning). The cell cycle time consists of two time intervals: the time interval before gene duplication and the time after gene duplication. These time intervals are modeled by using two independent phase-type distributions as shown in Figure B. Based on phase-type characteristics mean of the states of the first phase-type $\overline{\langle s_{ij} \rangle}$ and the second phase-type

$\overline{\langle g_{ij}\rangle}$ are

$$\overline{\langle s_{ij}\rangle} = \frac{p_i}{i}\beta, \quad i \in \{1, \ldots, n_1\}, \quad j \in \{1, \ldots, i\},$$
$$\overline{\langle g_{ij}\rangle} = \frac{p'_i}{i}(1-\beta), \quad i \in \{1, \ldots, n_2\}, \quad j \in \{1, \ldots, i\}, \tag{F.1}$$

where β is defined as

$$\beta := \frac{\text{Mean time interval before gene duplication}}{\text{Mean cell cycle time}} = \frac{\langle T_1\rangle}{\langle T\rangle}. \tag{F.2}$$

We start our analysis by deriving mean level of protein in the next section.

F.1 Mean of protein count level in the presence of gene duplication

After gene duplication the amount of genes expressing a specific protein doubles. Thus the rate of protein production increases by a factor f as shown in Figure A.A. This model coupled with phase-type distributions contains the following stochastic events

Event	Reset	Propensity
Protein production	$x(t) \mapsto x(t)+u$	$k_x p''_u \left(1+(f-1)\sum_{i=1}^{n_2}\sum_{j=1}^{i} g_{ij}\right)$
First phase-type evolution	$s_{ij}(t) \mapsto s_{ij}(t)-1$, $s_{i(j+1)}(t) \mapsto s_{i(j+1)}(t)+1$	$ik_1 s_{ij}$, $i \in \{2,\ldots,n_1\}$, $j \in \{1,\ldots,i-1\}$
Gene-duplication	$s_{jj}(t) \mapsto 0$, $g_{i1}(t) \mapsto g_{i1}(t)+1$	$k_1 p'_i \sum_{j=1}^{n_1} js_{jj}$, $i \in \{1,\ldots,n_2\}$
Second phase-type evolution	$g_{ij}(t) \mapsto g_{ij}(t)-1$, $g_{i(j+1)}(t) \mapsto g_{i(j+1)}(t)+1$	$ik_2 g_{ij}$, $i \in \{2,\ldots,n_2\}$, $j \in \{1,\ldots,i-1\}$
Cell-division	$x(t_s) \mapsto x_+(t_s)$, $g_{jj}(t_s) \mapsto 0$, $s_{i1}(t_s) \mapsto s_{i1}(t_s)+1$	$k_2 p_i \sum_{j=1}^{n_2} jg_{jj}$, $i \in \{1,\ldots,n_1\}$

Note that in the protein production event, before gene duplication all the states g_{ij} are zero thus propensity function will be $k_x p''_u$. After gene duplication and before division, one of the states g_{ij} is one hence propensity function will be $fk_x p''_u$. In time of gene duplication, states of the first phase-type will reset to zero and state g_{i1} of the second distribution will be selected with probability p'_i; hence propensity function of gene duplication event is $k_1 p'_i \sum_{j=1}^{n_1}(js_{jj})$. At the end of cell cycle, states of the second phase-type will reset to zero and a new cell cycle which is sum of i exponentials will be selected with probability p_i; thus propensity function of cell division event is $k_2 p_i \sum_{j=1}^{n_1}(jg_{jj})$.

Theorem 1 of [3] gives the time derivative of the expected value of any function $\varphi(x, s_{ij}, g_{ij})$ as

$$\frac{d\langle\varphi(x, s_{ij}, g_{ij})\rangle}{dt} = \left\langle \sum_{Events} \Delta\varphi(x, s_{ij}, g_{ij}) \times \psi(x, s_{ij}, g_{ij}) \right\rangle, \tag{F.3}$$

where $\Delta\varphi(x, s_{ij}, g_{ij})$ is a change in φ when an event occurs. The first-order moment dynamic of this model can be expressed by selecting φ to be x in equation (F.3)

$$\frac{d\langle x\rangle}{dt} = k_x\langle B\rangle \left(1 + (f-1)\left\langle \sum_{i=1}^{n_2}\sum_{j=1}^{i} g_{ij} \right\rangle\right) + k_2 \sum_{j=1}^{n_2} \left(j \left\langle (\frac{x}{2} - x) g_{jj} \right\rangle\right), \tag{F.4}$$

where conditional expected value of x_+ is replaced from equation (5).

Mean dynamics is not closed thus we add dynamics of $\langle xs_{ij}\rangle$, $i = \{1, \dots, n_1\}$, $j = \{1, \dots, i\}$ and $\langle xg_{ij}\rangle$, $i = \{1, \dots, n_1\}$, $j = \{1, \dots, i\}$ to have a closed set of moment equations. These moment dynamics are simplified by using equations (5), (9), (10) and

(F.1) as

$$\frac{d\langle xs_{i1}\rangle}{dt} = k_x\langle B\rangle\langle s_{i1}\rangle + \frac{k_2}{2}p_i'\sum_{j=1}^{n_2}\left(j\left\langle xg_{jj}\right\rangle\right) - ik_1\langle xs_{i1}\rangle, \tag{F.5a}$$

$$\frac{d\langle xs_{ij}\rangle}{dt} = k_x\langle B\rangle\langle s_{ij}\rangle - ik_1\langle xs_{ij}\rangle + ik_1\langle xs_{i(j-1)}\rangle, \quad j = \{2, \ldots, i\}, \tag{F.5b}$$

$$\frac{d\langle xg_{i1}\rangle}{dt} = fk_x\langle B\rangle\langle g_{i1}\rangle + k_1 p_i\sum_{j=1}^{n_1}\left(j\left\langle xs_{jj}\right\rangle\right) - ik_2\langle xg_{i1}\rangle, \tag{F.5c}$$

$$\frac{d\langle xg_{ij}\rangle}{dt} = fk_x\langle B\rangle\langle g_{ij}\rangle - ik_2\langle xg_{ij}\rangle + ik_2\langle xg_{i(j-1)}\rangle,\ j = \{2, \ldots, i\}. \tag{F.5d}$$

In order to find the mean of protein, first we need to find the moments $\overline{\langle xs_{ij}\rangle},\ i = \{1, \ldots, n_1\},\ j = \{1, \ldots, i\}$ and $\overline{\langle xg_{ij}\rangle},\ i = \{1, \ldots, n_2\},\ j = \{1, \ldots, i\}$. For calculating these moments we should calculate the term $\sum_{j=1}^{n_2}\left(j\overline{\langle xg_{jj}\rangle}\right)$; this term can be obtained by analyzing equation (F.4) in steady-state

$$k_x\langle B\rangle\left(f(1-\beta)+\beta\right) = \frac{k_2}{2}\sum_{j=1}^{n_2}\left(j\overline{\langle xg_{jj}\rangle}\right) \Rightarrow \sum_{j=1}^{n_2}\left(j\overline{\langle xg_{jj}\rangle}\right) = \frac{2k_x\langle B\rangle\left(f(1-\beta)+\beta\right)}{k_2}. \tag{F.6}$$

By having this term, we calculate $\overline{\langle xs_{ij}\rangle}$ by recursion process: we start by calculating $\overline{\langle xs_{i1}\rangle}$ by substituting equation (F.6) in equation (F.5a). In the next step we use the definition we derived for $\overline{\langle xs_{i1}\rangle}$ to calculate $\overline{\langle xs_{i2}\rangle}$ from equation (F.5b). We continue this process until we derive all the moments

$$\overline{\langle xs_{ij}\rangle} = \frac{k_x\langle B\rangle}{ik_1}p_i'\left(\beta\frac{j}{i} + \left(f(1-\beta)+\beta\right)\right),\ i = \{1, \ldots, n_1\},\ j = \{1, \ldots, i\}. \tag{F.7}$$

Now we need to calculate the moments $\overline{\langle xg_{ij}\rangle},\ i = \{1, \ldots, n_2\},\ j = \{1, \ldots, i\}$, thus we

need the expression of the term $\sum_{j=1}^{n_1}\left(j\overline{\langle xs_{jj}\rangle}\right)$; from equation (F.7) we have the following

$$\sum_{j=1}^{n_1}\left(j\overline{\langle xs_{jj}\rangle}\right)=\frac{k_x\langle B\rangle\left(f(1-\beta)+2\beta\right)}{k_1}. \tag{F.8}$$

Substituting this term in equations (F.5c) and (F.5d) result in

$$\overline{\langle xg_{ij}\rangle}=\frac{fk_x\langle B\rangle}{ik_2}p_i\left((1-\beta)\frac{j}{i}+(1-\beta)\right)+\frac{2\beta k_x\langle B\rangle}{ik_2}p_i,\ i=\{1,\ldots,n_2\},\ j=\{1,\ldots,i\}. \tag{F.9}$$

Note that

$$\begin{aligned}\sum_{i=1}^{n_1}\sum_{j=1}^{i}s_{ij}+\sum_{i=1}^{n_2}\sum_{j=1}^{i}g_{ij}=1\Rightarrow\langle x\rangle&=\left\langle x\left(\sum_{i=1}^{n_1}\sum_{j=1}^{i}s_{ij}+\sum_{i=1}^{n_2}\sum_{j=1}^{i}g_{ij}\right)\right\rangle\\ \Rightarrow\overline{\langle x\rangle}&=\sum_{i=1}^{n_1}\sum_{j=1}^{i}\overline{\langle xs_{ij}\rangle}+\sum_{i=1}^{n_2}\sum_{j=1}^{i}\overline{\langle xg_{ij}\rangle}.\end{aligned} \tag{F.10}$$

Thus by adding all the term calculated here and using equation (7) mean of protein can be calculated as

$$\overline{\langle x\rangle}=\frac{k_x\langle B\rangle\langle T_1\rangle\left(2f(1-\beta)+3\beta+\beta CV_{T_1}^2\right)}{2}+\frac{k_x\langle B\rangle\langle T_2\rangle\left(3f(1-\beta)+4\beta+f(1-\beta)CV_{T_2}^2\right)}{2}. \tag{F.11}$$

F.2 Noise in protein count level contributed from cell cycle time variations

In order to calculate the noise contributed from cell cycle time variation, the model introduced in Figure A.B coupled with phase-type distributions is used. This model contains following stochastic events

Event	Reset	Propensity
First phase-type evolution	$s_{ij}(t) \mapsto s_{ij}(t) - 1$, $s_{i(j+1)}(t) \mapsto s_{i(j+1)}(t) + 1$	$ik_1 s_{ij}$, $i \in \{2, \ldots, n_1\}$, $j \in \{1, \ldots, i-1\}$
Gene-duplication	$s_{jj}(t) \mapsto 0$, $g_{i1}(t) \mapsto g_{i1}(t) + 1$	$k_1 p'_i \sum_{j=1}^{n_1} (js_{jj})$, $i \in \{1, \ldots, n_2\}$
Second phase-type evolution	$g_{ij}(t) \mapsto g_{ij}(t) - 1$, $g_{i(j+1)}(t) \mapsto g_{i(j+1)}(t) + 1$	$ik_2 g_{ij}$, $i \in \{2, \ldots, n_2\}$, $j \in \{1, \ldots, i-1\}$
Cell-division	$x(t_s) \mapsto x(t_s)/2$, $g_{jj}(t_s) \mapsto 0$, $s_{i1}(t_s) \mapsto s_{i1}(t_s) + 1$	$k_2 p_i \sum_{j=1}^{n_2} (jg_{jj})$, $i \in \{1, \ldots, n\}$

and deterministic protein production

$$\dot{x} = k_x \langle B \rangle \left(1 + (f-1) \sum_{i=1}^{n_2} \sum_{j=1}^{i} g_{ij} \right). \tag{F.12}$$

Theorem 1 of [3] gives the time derivative of the expected value of any function

$\varphi(x, s_{ij}, g_{ij})$ as

$$\frac{d\langle\varphi(x,s_{ij},g_{ij})\rangle}{dt} = \left\langle \sum_{Events} \Delta\varphi(x,s_{ij},g_{ij}) \times \psi(x,s_{ij},g_{ij}) \right\rangle + \left\langle \frac{\partial\varphi(x,g_{ij})}{\partial x} k_x\langle B\rangle \left(1+(f-1)\sum_{i=1}^{n_2}\sum_{j=1}^{i} g_{ij}\right)\right\rangle, \tag{F.13}$$

where the first term in the right hand side is contributed from stochastic events, and the second term is contributed from deterministic protein production. In this model, dynamics of $\langle x\rangle$, $\langle xs_{ij}\rangle$ and $\langle xg_{ij}\rangle$ are the same as equations (F.4) and (E.6), thus mean of protein, $\overline{\langle xs_{ij}\rangle}$, and $\overline{\langle xg_{ij}\rangle}$ will be equal to their value in previous section. Further, the second-order moment dynamics of protein can be added by selecting φ to be x^2 in equation (F.13)

$$\frac{d\langle x^2\rangle}{dt} = 2k_x\langle B\rangle \left(\langle x\rangle + (f-1)\left\langle \sum_{i=1}^{n_2}\sum_{j=1}^{i} xg_{ij}\right\rangle\right) - \frac{3k_2}{4}\sum_{j=1}^{n_2}\left(j\left\langle x^2 g_{jj}\right\rangle\right). \tag{F.14}$$

This equation is not closed thus we add dynamics of $\langle x^2 s_{ij}\rangle,\ i=\{1,\dots,n_1\},\ j=\{1,\dots,i\}$ and $\langle x^2 g_{ij}\rangle,\ i=\{1,\dots,n_2\},\ j=\{1,\dots,i\}$ to have a closed set of equations

$$\frac{d\langle x^2 s_{i1}\rangle}{dt} = 2k_x\langle B\rangle\langle xs_{i1}\rangle + \frac{k_2}{4}p_i\sum_{j=1}^{n_2}\left(j\left\langle x^2 g_{jj}\right\rangle\right) - ik_1\langle x^2 s_{i1}\rangle, \tag{F.15a}$$

$$\frac{d\langle x^2 s_{ij}\rangle}{dt} = 2k_x\langle B\rangle\langle xs_{ij}\rangle - ik_1\langle x^2 s_{ij}\rangle + ik_1\langle x^2 s_{(i-1)j}\rangle,\ j=\{2,\dots,i\}, \tag{F.15b}$$

$$\frac{d\langle x^2 g_{i1}\rangle}{dt} = 2fk_x\langle B\rangle\langle xg_{i1}\rangle + k_1p_i\sum_{j=1}^{n_1}\left(j\left\langle x^2 s_{jj}\right\rangle\right) - ik_2\langle x^2 g_{i1}\rangle, \tag{F.15c}$$

$$\frac{d\langle x^2 g_{ij}\rangle}{dt} = 2fk_x\langle B\rangle\langle xg_{ij}\rangle - ik_2\langle x^2 g_{ij}\rangle + ik_2\langle x^2 g_{(i-1)j}\rangle, j=\{2,\dots,i\}. \tag{F.15d}$$

In order to calculate noise we need to express $\overline{\langle x^2 s_{ij}\rangle}$, and $\overline{\langle x^2 g_{ij}\rangle}$, which requires calcu-

lating the term $\sum_{j=1}^{n_2}\left(j\overline{\langle x^2 g_{jj}\rangle}\right)$; this term can be derived by analyzing equation (F.14) in steady-state

$$\begin{aligned}\frac{3k_2}{4}\sum_{j=1}^{n_2}\left(j\overline{\langle x^2 g_{jj}\rangle}\right) =& 2k_x\langle B\rangle\left(\overline{\langle x\rangle} + (f-1)\overline{\left\langle\sum_{i=1}^{n_2}\sum_{j=1}^{i} x g_{ij}\right\rangle}\right) \Rightarrow \\ \sum_{j=1}^{n_2}\left(j\overline{\langle x^2 g_{jj}\rangle}\right) =& \frac{4k_x^2\langle B\rangle^2\langle T_1\rangle\left(2f(1-\beta)+3\beta+\beta CV_{T_1}^2\right)}{3k_2} \\ &+ \frac{4fk_x^2\langle B\rangle^2\langle T_2\rangle\left(3f(1-\beta)+4\beta+f(1-\beta)CV_{T_2}^2\right)}{3k_2},\end{aligned} \tag{F.16}$$

where in deriving this term we used equation (F.11) and we summed all the terms in equation (F.9). By having this term, we calculate $\overline{\langle x^2 s_{ij}\rangle}$ by recursion process. we derive $\overline{\langle x^2 s_{i1}\rangle}$ by substituting equation (F.16) in equation (F.15a). In the next step we use the definition of $\overline{\langle x^2 s_{i1}\rangle}$ to calculate $\overline{\langle x^2 s_{i2}\rangle}$ from equation (F.15b). We continue this process until we derive all the moments

$$\begin{aligned}\overline{\langle x^2 s_{ij}\rangle} =& \frac{k_x^2\langle B\rangle^2\langle T_1\rangle\left(2f(1-\beta)+3\beta+\beta CV_{T_1}^2\right)}{3ik_1}p_i' \\ &+ \frac{fk_x^2\langle B\rangle^2\langle T_2\rangle\left(3f(1-\beta)+4\beta+f(1-\beta)CV_{T_2}^2\right)}{3ik_1}p_i' \\ &+ \frac{k_x^2\langle B\rangle^2}{i^2k_1^2}p_i'\left(\frac{\beta j^2+2(f(1-\beta)+\beta)ij+\beta j}{i}\right),\ i=\{1,\dots,n_1\},\ j=\{1,\dots,i\}.\end{aligned} \tag{F.17}$$

Expressing $\overline{\langle x^2 g_{ij}\rangle}$ requires calculation of the term $\sum_{j=1}^{n_1}\left(j\overline{\langle x^2 s_{jj}\rangle}\right)$ which can be obtained from equation (F.17) as

$$\begin{aligned}&\sum_{j=1}^{n_1}\left(j\overline{\langle x^2 s_{jj}\rangle}\right) = \\ &\frac{4k_x^2\langle B\rangle^2\langle T_1\rangle\left(2f(1-\beta)+3\beta+\beta CV_{T_1}^2\right)}{3k_1} + \frac{fk_x^2\langle B\rangle^2\langle T_2\rangle\left(3f(1-\beta)+4\beta+f(1-\beta)CV_{T_2}^2\right)}{3k_1}.\end{aligned} \tag{F.18}$$

Thus $\overline{\langle x^2 g_{ij}\rangle}$ can be obtained with a recursion process from equations (F.15c) and (F.15d)

$$
\begin{aligned}
\overline{\langle x^2 g_{ij}\rangle} =& \frac{4k_x^2\langle B\rangle^2\langle T_1\rangle\left(2f(1-\beta)+3\beta+\beta CV_{T_1}^2\right)}{3ik_2}p_i \\
&+ \frac{fk_x^2\langle B\rangle^2\langle T_2\rangle\left(3f(1-\beta)+4\beta+f(1-\beta)CV_{T_2}^2\right)}{3ik_2}p_i \\
&+ \frac{f^2k_x^2\langle B\rangle^2}{ik_2}p_i\left(\frac{(1-\beta)j^2+(1-\beta)j+2(1-\beta)ij}{i}\right)+\frac{4fk_x^2\langle B\rangle^2}{ik_2}p_i\left(\frac{ij}{i}\right), \\
& i=\{1,\ldots,n_2\},\ j=\{1,\ldots,i\}.
\end{aligned} \tag{F.19}
$$

Note that $\sum_{i=1}^{n_1}\sum_{j=1}^{i}\overline{\langle x^2 s_{ij}\rangle}+\sum_{i=1}^{n_2}\sum_{j=1}^{i}\overline{\langle x^2 g_{ij}\rangle}=\overline{\langle x^2\rangle}$ thus the second order moment of protein can be derived by adding all the terms in equations (F.17) and (F.19). $\overline{\langle x^2\rangle}$ can be simplified by using equations (7) in the main article as

$$
\begin{aligned}
&\overline{\langle x^2\rangle} = k_x^2\langle B\rangle^2\left(\frac{\langle T_1^3\rangle+f^2\langle T_2^3\rangle}{3\langle T\rangle}+\right. \\
&\left.\langle T\rangle^3\frac{2\beta^2(-3\beta+2CV_{T_1}^2+6)+3\beta(\beta-1)f(3\beta-\beta CV_{T_1}^2+2(\beta-1)CV_{T_2}^2-6)+(\beta-1)^2\left(-f^2\right)(3\beta+3\beta CV_{T_2}^2-4CV_{T_2}^2-6)}{3\langle T\rangle}\right).
\end{aligned} \tag{F.20}
$$

Finally, using the definition of CV^2 results in noise of protein raised from cell cycle time variations

$$
\begin{aligned}
CV_E^2 =& \frac{(4\langle T_1^3\rangle+4f^2\langle T_2^3\rangle)/\langle T\rangle^3}{3(\beta(\beta(CV_{T_1}^2-1)+4)+(\beta-1)f(\beta+(\beta-1)CV_{T_2}^2-3))^2} \\
&+ \frac{\beta^2\left(-3\beta^2(1-CV_{T_1}^2)^2-24\beta CV_{T_1}^2-6(\beta-1)^2(CV_{T_1}^2-1)(CV_{T_2}^2+1)f+16CV_{T_1}^2\right)}{3(\beta(\beta(CV_{T_1}^2-1)+4)+(\beta-1)f(\beta+(\beta-1)CV_{T_2}^2-3))^2} \\
&- \frac{(\beta-1)^2f^2\left(3CV_{T_2}^4+3\beta^2(CV_{T_2}^2+1)^2-6\beta(CV_{T_2}^2+1)^2+2CV_{T_2}^2+3\right)}{3(\beta(\beta(CV_{T_1}^2-1)+4)+(\beta-1)f(\beta+(\beta-1)CV_{T_2}^2-3))^2}.
\end{aligned} \tag{F.21}
$$

F.3 Noise in protein count level contributed from random partitioning

In order to take into account noise caused by random partitioning of proteins between two daughter cells, we use the model shown in Figure A.C coupled with phase-type distributions. This model contains the following stochastic events

Event	Reset	Propensity
First phase-type evolution	$s_{ij}(t) \mapsto s_{ij}(t)-1,$ $s_{i(j+1)}(t) \mapsto s_{i(j+1)}(t)+1$	$ik_1 s_{ij},$ $i \in \{2,\dots,n_1\},$ $j \in \{1,\dots,i-1\}$
Gene-duplication	$s_{jj}(t) \mapsto 0,$ $g_{i1}(t) \mapsto g_{i1}(t)+1$	$k_1 p'_i \sum_{j=1}^{n_1}(js_{jj}),$ $i \in \{1,\dots,n_2\}$
Second phase-type evolution	$g_{ij}(t) \mapsto g_{ij}(t)-1,$ $g_{i(j+1)}(t) \mapsto g_{i(j+1)}(t)+1$	$ik_2 g_{ij},$ $i \in \{2,\dots,n_2\},$ $j \in \{1,\dots,i-1\}$
Cell-division	$x(t_s) \mapsto x_+(t_s),$ $g_{jj}(t_s) \mapsto 0,$ $s_{i1}(t_s) \mapsto s_{i1}(t_s)+1$	$k_2 p_i \sum_{j=1}^{n_2}(jg_{jj}),$ $i \in \{1,\dots,n\}$

and deterministic protein production

$$\dot{x} = k_x \langle B \rangle \left(1 + (f-1)\sum_{i=1}^{n_2}\sum_{j=1}^{i} g_{ij}\right). \tag{F.22}$$

Note that here x is a continuous random variable, hence x_+ is also obtained from a continious distribution. Connection between statistical statistical moments of x and x_+ is given by (5).

For this model, $\overline{\langle x \rangle}$, $\overline{\langle xs_{ij} \rangle}$, and $\overline{\langle xg_{ij} \rangle}$ are equal to their value in Section E.1 and

Section E.2. However, dynamics of $\langle x^2\rangle$ and $\langle x^2 s_{i1}\rangle$ are different

$$\frac{d\langle x^2\rangle}{dt} = 2k_x\langle B\rangle\left(\langle x\rangle + (f-1)\left\langle\sum_{i=1}^{n_2}\sum_{j=1}^{i} xg_{ij}\right\rangle\right) + \frac{1}{4}\alpha k_2\sum_{j=1}^{n_2}\left(j\left\langle xg_{jj}\right\rangle\right) - \frac{3k_2}{4}\sum_{j=1}^{n_2}\left(j\left\langle x^2 g_{jj}\right\rangle\right), \tag{F.23a}$$

$$\frac{d\langle x^2 s_{i1}\rangle}{dt} = 2k_x\langle B\rangle\langle xs_{i1}\rangle + \frac{k_2}{4}p_i\sum_{j=1}^{n_2}\left(j\left\langle x^2 g_{jj}\right\rangle\right) + \frac{1}{4}\alpha k_2\sum_{j=1}^{n_2}\left(j\left\langle xg_{jj}\right\rangle\right) - ik_1\langle x^2 s_{i1}\rangle, \tag{F.23b}$$

note that dynamics of $\langle x^2 s_{ij}\rangle$, $j=\{2,\ldots,i\}$ and $\langle x^2 g_{ij}\rangle$ are identical to equations (F.15b), (F.15c), and (F.15d). Similar to previous section, we derive $\overline{\langle x^2 s_{ij}\rangle}$ and $\overline{\langle x^2 g_{ij}\rangle}$ as

$$\begin{aligned}\overline{\langle x^2 s_{ij}\rangle} =& \frac{k_x^2\langle B\rangle^2\langle T_1\rangle\left(2f(1-\beta)+3\beta+\beta CV_{T_1}^2\right)}{3ik_1}p_i' \\ &+ \frac{fk_x^2\langle B\rangle^2\langle T_2\rangle\left(3f(1-\beta)+4\beta+f(1-\beta)CV_{T_2}^2\right)}{3ik_1}p_i' \\ &+ \frac{k_x^2\langle B\rangle^2}{i^2k_1^2}p_i'\left(\frac{\beta j^2+2(f(1-\beta)+\beta)ij+\beta j}{i}\right) + \frac{2\alpha k_x\langle B\rangle(f(1-\beta)+\beta)}{3ik_1}p_i', \\ & i=\{1,\ldots,n_1\}\ j=\{1,\ldots,i\}.\end{aligned} \tag{F.24}$$

$$\begin{aligned}\overline{\langle x^2 g_{ij}\rangle} =& \frac{4k_x^2\langle B\rangle^2\langle T_1\rangle\left(2f(1-\beta)+3\beta+\beta CV_{T_1}^2\right)}{3ik_2}p_i \\ &+ \frac{fk_x^2\langle B\rangle^2\langle T_2\rangle\left(3f(1-\beta)+4\beta+f(1-\beta)CV_{T_2}^2\right)}{3ik_2}p_i \\ &+ \frac{f^2k_x^2\langle B\rangle^2}{ik_2}p_i\left(\frac{(1-\beta)j^2+(1-\beta)j+2(1-\beta)ij}{i}\right) + \frac{4fk_x^2\langle B\rangle^2}{ik_2}p_i\left(\frac{ij}{i}\right) \\ &+ \frac{2\alpha k_x\langle B\rangle(f(1-\beta)+\beta)}{3ik_2}p_i,\quad i=\{1,\ldots,n_2\},\ j=\{1,\ldots,i\}.\end{aligned} \tag{F.25}$$

Hence the second-order moment is

$$\overline{\langle x^2\rangle} = k_x^2\langle B\rangle^2\left(\frac{\langle T_1^3\rangle + f^2\langle T_2^3\rangle}{3\langle T\rangle} + \langle T\rangle^3\frac{2\beta^2(-3\beta+2CV_{T_1}^2+6)+3\beta(\beta-1)f(3\beta-\beta CV_{T_1}^2+2(\beta-1)CV_{T_2}^2-6)+(\beta-1)^2\left(-f^2\right)(3\beta+3\beta CV_{T_2}^2-4CV_{T_2}^2-6)}{3\langle T\rangle}\right) + \frac{2\alpha k_x\langle B\rangle(f(1-\beta)+\beta)\langle T\rangle}{3}. \tag{F.26}$$

Squared coefficient of variation gives noise raised from partitioning and cell cycle variations, which subtracting equation (F.21) from results gives partitioning noise as

$$CV_R^2 = \frac{4\alpha(f(1-\beta)+\beta)}{3\left((\beta^2(f-1)-4\beta(f-1)+3f)+\beta^2CV_{T_1}^2+f(1-\beta)^2CV_{T_2}^2\right)}\frac{1}{\overline{\langle x\rangle}}. \tag{F.27}$$

F.4 Noise in protein count level contributed from stochastic production

In order to calculate the noise caused by stochastic birth of protein, we use the model introduced in Section C.1. For this model, moments dynamics of $\langle x^2\rangle$, $\langle x^2 s_{ij}\rangle$, and $\langle x^2 g_{ij}\rangle$ can be written as

$$\begin{aligned}\frac{d\langle x^2\rangle}{dt} =& k_x\langle B^2\rangle\left(1+(f-1)\left\langle\sum_{i=1}^{n_2}\sum_{j=1}^{i} g_{ij}\right\rangle\right)+2k_x\langle B\rangle\left(\langle x\rangle+(f-1)\left\langle\sum_{i=1}^{n_2}\sum_{j=1}^{i} xg_{ij}\right\rangle\right)\\&+\frac{1}{4}\alpha k_2\sum_{j=1}^{n_2}\left(j\left\langle xg_{jj}\right\rangle\right)-\frac{3k_2}{4}\sum_{j=1}^{n_2}\left(j\left\langle x^2 g_{jj}\right\rangle\right),\end{aligned} \tag{F.28a}$$

$$\begin{aligned}\frac{d\langle x^2 s_{i1}\rangle}{dt} =& k_x\langle B^2\rangle\langle s_{i1}\rangle+2k_x\langle B\rangle\langle xs_{i1}\rangle+\frac{k_2}{4}p_i\sum_{j=1}^{n_2}\left(j\left\langle x^2 g_{jj}\right\rangle\right)\\&+\frac{1}{4}\alpha k_2\sum_{j=1}^{n_2}\left(j\left\langle xg_{jj}\right\rangle\right)-ik_1\langle x^2 s_{i1}\rangle,\end{aligned} \tag{F.28b}$$

$$\frac{d\langle x^2 s_{ij}\rangle}{dt} = k_x\langle B^2\rangle\langle s_{ij}\rangle+2k_x\langle B\rangle\langle xs_{ij}\rangle-ik_1\langle x^2 s_{ij}\rangle+ik_1\langle x^2 s_{(i-1)j}\rangle,\ j=\{2,\ldots,i\}, \tag{F.28c}$$

$$\frac{d\langle x^2 g_{i1}\rangle}{dt} = fk_x\langle B^2\rangle\langle g_{i1}\rangle+2fk_x\langle B\rangle\langle xg_{i1}\rangle+k_1p_i\sum_{j=1}^{n_1}\left(j\left\langle x^2 s_{jj}\right\rangle\right)-ik_2\langle x^2 g_{i1}\rangle, \tag{F.28d}$$

$$\frac{d\langle x^2 g_{ij}\rangle}{dt} = fk_x\langle B^2\rangle\langle g_{ij}\rangle+2fk_x\langle B\rangle\langle xg_{ij}\rangle-ik_2\langle x^2 g_{ij}\rangle+ik_2\langle x^2 g_{(i-1)j}\rangle,\ j=\{2,\ldots,i\}. \tag{F.28e}$$

The same as before we derive $\overline{\langle x^2 s_{ij}\rangle}$

$$\begin{aligned}\overline{\langle x^2 s_{ij}\rangle} =& \frac{k_x^2\langle B\rangle^2\langle T_1\rangle\left(2f(1-\beta)+3\beta+\beta CV_{T_1}^2\right)}{3ik_1}p_i'\\&+\frac{fk_x^2\langle B\rangle^2\langle T_2\rangle\left(3f(1-\beta)+4\beta+f(1-\beta)CV_{T_2}^2\right)}{3ik_1}p_i'\\&+\frac{k_x^2\langle B\rangle^2}{i^2k_1^2}p_i'\left(\frac{\beta j^2+2(f(1-\beta)+\beta)ij+\beta j}{i}\right)+\frac{2\alpha k_x\langle B\rangle(f(1-\beta)+\beta)}{3ik_1}p_i'\\&+\frac{k_x\langle B^2\rangle}{ik_1}\left(\frac{f(1-\beta)+\beta}{3}+\beta\frac{j}{i}\right)p_i',\ i=\{1,\ldots,n_1\},\ j=\{1,\ldots,i\},\end{aligned} \tag{F.29}$$

and $\overline{\langle x^2 g_{ij}\rangle}$

$$\begin{aligned}\overline{\langle x^2 g_{ij}\rangle} =& \frac{4k_x^2\langle B\rangle^2\langle T_1\rangle\left(2f(1-\beta)+3\beta+\beta CV_{T_1}^2\right)}{3ik_2}p_i \\ &+ \frac{fk_x^2\langle B\rangle^2\langle T_2\rangle\left(3f(1-\beta)+4\beta+f(1-\beta)CV_{T_2}^2\right)}{3ik_2}p_i \\ &+ \frac{f^2k_x^2\langle B\rangle^2}{ik_2}p_i\left(\frac{(1-\beta)j^2+(1-\beta)j+2(1-\beta)ij}{i}\right)+\frac{4fk_x^2\langle B\rangle^2}{ik_2}p_i\left(\frac{ij}{i}\right) \\ &+ \frac{2\alpha k_x\langle B\rangle(f(1-\beta)+\beta)}{3ik_2}p_i+\frac{k_x\langle B^2\rangle}{ik_2}\left(\frac{f(1-\beta)+4\beta}{3}+f(1-\beta)\frac{j}{i}\right)p_i \\ & i=\{1,\dots,n_2\},\ j=\{1,\dots,i\}.\end{aligned} \tag{F.30}$$

Finally summing all the moments $\overline{\langle x^2 s_{ij}\rangle}$, and $\overline{\langle x^2 g_{ij}\rangle}$ results in $\overline{\langle x^2\rangle}$ as

$$\begin{aligned}\overline{\langle x^2\rangle} =& k_x^2\langle B\rangle^2\left(\frac{\langle T_1^3\rangle+f^2\langle T_2^3\rangle}{3\langle T\rangle}+\right. \\ &\left.\langle T\rangle^3\frac{2\beta^2(-3\beta+2CV_{T_1}^2+6)+3\beta(\beta-1)f(3\beta-\beta CV_{T_1}^2+2(\beta-1)CV_{T_2}^2-6)+(\beta-1)^2\left(-f^2\right)(3\beta+3\beta CV_{T_2}^2-4CV_{T_2}^2-6)}{3\langle T\rangle}\right) \\ &+\frac{2\alpha k_x\langle B\rangle(f(1-\beta)+\beta)\langle T\rangle}{3}+k_x\langle B^2\rangle\left(\frac{f(1-\beta)+\beta}{3}+\beta\left(\frac{1+CV_{T_1}^2}{2}\right)\right)\langle T_1\rangle \\ &+k_x\langle B^2\rangle\left(\frac{f(1-\beta)+4\beta}{3}+f(1-\beta)\left(\frac{1+CV_{T_2}^2}{2}\right)\right)\langle T_2\rangle.\end{aligned} \tag{F.31}$$

Steady-state analysis gives the noise from stochastic birth, random partitioning, and cell cycle time variations. Subtracting noise of cell cycle time and partitioning in equations (F.21) and (F.27) results in noise caused by stochastic production of protein

$$CV_P^2 = \frac{(5f-8\beta(f-1)+3\beta^2(f-1))+3f(1-\beta)^2CV_{T_2}^2+3\beta^2CV_{T_1}^2}{3\left((\beta^2(f-1)-4\beta(f-1)+3f)+\beta^2CV_{T_1}^2+f(1-\beta)^2CV_{T_2}^2\right)}\frac{\langle B^2\rangle}{\langle B\rangle}\frac{1}{\overline{\langle x\rangle}}. \tag{F.32}$$

F.5 Effect of gene duplication time on intrinsic noise

We investigate how the noise contributions from random partitioning and stochastic expression (CV_R^2 and CV_P^2 terms in equation (45) of the main text) change as β is varied between 0 and 1 for $f = 2$. Results show that CV_R^2 and CV_P^2 follow the same qualitative shapes as reported in Figure 6. There exists a β^*

$$\beta^* = \frac{-\sqrt{2(2CV_{T_1}^4 + 5CV_{T_1}^2 CV_{T_2}^2 + 3CV_{T_1}^2 + 2CV_{T_2}^4 + 3CV_{T_2}^2 + 1)} + 2CV_{T_1}^2 + 4CV_{T_2}^2 + 2}{CV_{T_1}^2 + 2CV_{T_2}^2 + 1}, \tag{F.33}$$

such that CV_P^2 is minimized and CV_R^2 is maximized when $\beta = \beta^*$. Note that when $CV_{T_1}^2 = CV_{T_2}^2 = 0$, $\beta^* = 2 - \sqrt{2}$ as reported in the main text. The minimum value of CV_P^2 and the maximum value of CV_R^2 are given by

$$CV_P^2 = \frac{CV_{T_1}^2(3CV_{T_2}^2 + 7) - \sqrt{2(2CV_{T_1}^2 + CV_{T_2}^2 + 1)(CV_{T_1}^2 + 2CV_{T_2}^2 + 1)} + 7CV_{T_2}^2 + 3}{3(CV_{T_1}^2(CV_{T_2}^2 + 3) + 3CV_{T_2}^2 + 1)} \frac{\langle B^2 \rangle}{\langle B \rangle} \frac{1}{\langle x \rangle}, \tag{F.34}$$

$$CV_R^2 = \frac{\sqrt{2}\alpha}{3\sqrt{(2CV_{T_1}^2 + CV_{T_2}^2 + 1)(CV_{T_1}^2 + 2CV_{T_2}^2 + 1)} - 3\sqrt{2}CV_{T_1}^2 - 3\sqrt{2}CV_{T_2}^2}, \tag{F.35}$$

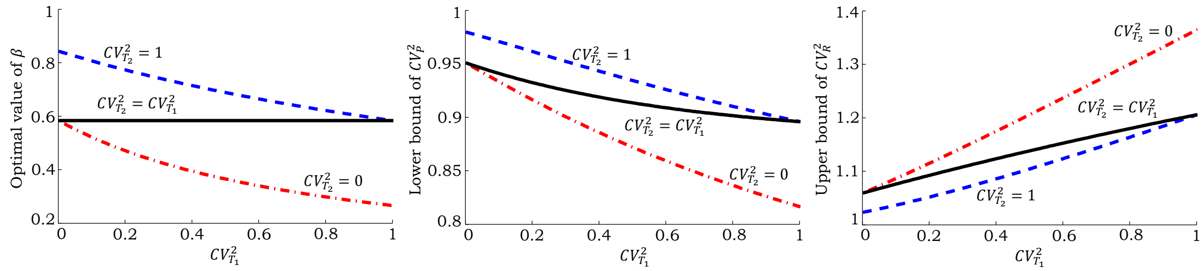

Figure C: **Effect of gene duplication on intrinsic noise level**. *Left*: Value of β where CV_P^2 is minimized and CV_R^2 is maximized as a function of $CV_{T_1}^2$. When $CV_{T_1}^2 = CV_{T_2}^2$, noise levels always reach their extrema at $\beta = 2 - \sqrt{2}$. *Middle & Right*: Extremum values of CV_P^2 and CV_R^2 as a functions of $CV_{T_1}^2$. Noise levels are normalized by their values at $\beta = 0$.

respectively. Plots of β^* and optimal value of CV_R^2 and CV_P^2 as a function of $CV_{T_1}^2$ are shown in Figure D. Note that if noise in T_1 is high and T_2 is deterministic then β^* shifts towards zero. Similarly, if noise in T_2 is high and T_1 is deterministic then β^* shifts towards one.

Appendix G

Noise level in unstable protein

Consider an unstable protein with sufficiently high degradation rate γ_x such that the protein level reaches steady-state instantaneously compared to the cell cycle time (Figure D). Let τ denote the time from the last division event, then

$$\overline{\langle x|\tau < T_1\rangle} = \frac{k_x\langle B\rangle}{\gamma_x}, \qquad \overline{\langle x|\tau > T_1\rangle} = \frac{fk_x\langle B\rangle}{\gamma_x}, \tag{G.1}$$

where T_1 is the time in which duplication happens. The mean level of an unstable protein can be calculated as

$$\overline{\langle x\rangle} = \overline{\langle x|\tau < T_1\rangle}p(\tau < T_1) + \overline{\langle x|\tau > T_1\rangle}p(\tau > T_1), \tag{G.2}$$

where $p(\tau < T_1)$ and $p(\tau > T_1)$ denote the probability of being in the time interval before and after gene duplication. Using

$$p(\tau < T_1) = \beta, \quad p(\tau > T_1) = (1-\beta), \tag{G.3}$$

we obtain

$$\overline{\langle x\rangle} = \frac{k_x\langle B\rangle(f(1-\beta)+\beta)}{\gamma_x}. \tag{G.4}$$

To compute the extrinsic noise component we consider deterministic protein produc-

tion and decay. The second-order moment of $x(t)$ is given by

$$\begin{matrix} \overline{\langle x^2|\tau < T_1\rangle} = \left(\frac{k_x\langle B\rangle}{\gamma_x}\right)^2 \\ \overline{\langle x^2|\tau > T_1\rangle} = \left(\frac{fk_x\langle B\rangle}{\gamma_x}\right)^2 \end{matrix} \Rightarrow \overline{\langle x^2\rangle} = \left(\frac{k_x\langle B\rangle}{\gamma_x}\right)^2\beta + \left(\frac{fk_x\langle B\rangle}{\gamma_x}\right)^2(1-\beta). \tag{G.5}$$

By using definition of CV^2, extrinsic noise is

$$CV_E^2 = \frac{(1-\beta)\beta(f-1)^2}{(\beta + f(1-\beta))^2}, \tag{G.6}$$

which is zero at $\beta = 0, 1$ and reaches its maximum at $\beta = f/(1+f)$ (Figure D).

Next we compute the intrinsic noise component. If the protein decay is sufficiently high, the noise contribution from partitioning errors will be negligible because any errors will be instantaneously corrected due to rapid protein turnover. Noise raised from stochastic gene expression can be investigated by considering a model containing stochastic bursty production and stochastic degradation of proteins, where after gene duplication the burst frequency doubles. Again assuming large enough γ_x, $\overline{\langle x^2|\tau < T_1\rangle}$ is equal to the steady-state second-order moment of a stochastic model with burst frequency k_x (analyzed in [4])

$$\overline{\langle x^2|\tau < T_1\rangle} = \left(\frac{k_x\langle B\rangle}{\gamma_x}\right)^2 + \frac{k_x\langle B^2\rangle}{2\gamma_x} + \frac{k_x\langle B\rangle}{2\gamma_x}. \tag{G.7}$$

In comparison with equation (G.5), there are two extra terms at the right hand side of $\overline{\langle x^2|\tau < T_1\rangle}$. The first extra term is due to production of protein in random bursts and the second one is due to stochastic degradation of protein molecules. Further for the same reasons (large degradation rate and rapid equilibration of the distribution), $\overline{\langle x^2|\tau > T_1\rangle}$ is equal to the second-order moment of a model containing stochastic bursty production

of proteins with burst frequency fk_x which is

$$\overline{\langle x^2|\tau > T_1\rangle} = \left(\frac{fk_x\langle B\rangle}{\gamma_x}\right)^2 + \frac{fk_x\langle B^2\rangle}{2\gamma_x} + \frac{fk_x\langle B\rangle}{2\gamma_x}. \tag{G.8}$$

Thus the second order moment of an unstable protein can be written as

$$\begin{aligned}\overline{\langle x^2\rangle} = &\left(\frac{k_x\langle B\rangle}{\gamma_x}\right)^2\beta + \frac{k_x\langle B^2\rangle}{2\gamma_x}\beta + \frac{k_x\langle B\rangle}{2\gamma_x}\beta \\ &+ \left(\frac{fk_x\langle B\rangle}{\gamma_x}\right)^2(1-\beta) + \frac{fk_x\langle B^2\rangle}{2\gamma_x}(1-\beta) + \frac{fk_x\langle B\rangle}{2\gamma_x}(1-\beta).\end{aligned} \tag{G.9}$$

Using definition of CV^2 and subtracting extrinsic noise we obtain the following noise contribution from stochastic expression and decay

$$CV_P^2 = \frac{1}{2}\left(\frac{\langle B^2\rangle}{\langle B\rangle} + 1\right)\frac{1}{\overline{\langle x\rangle}}. \tag{G.10}$$

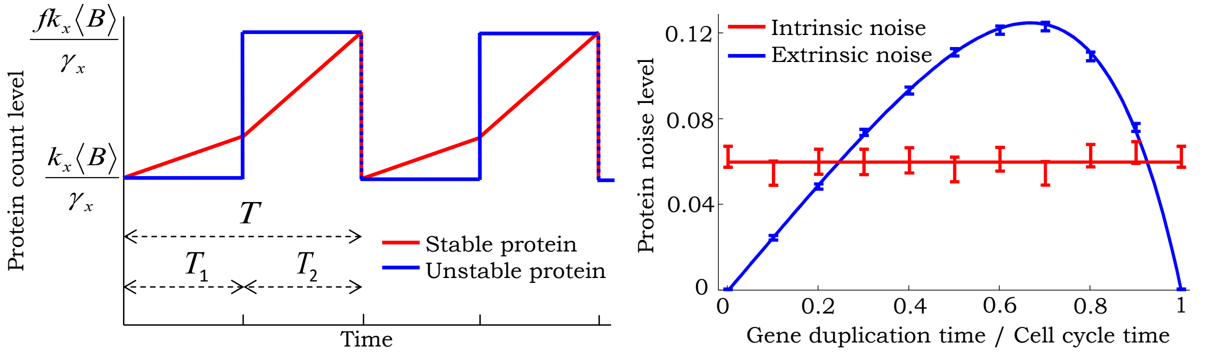

Figure D: **Contribution of gene duplication to noise levels of an unstable protein.** *Left*: For a stable protein, copy numbers accumulate in a bilinear fashion. In contrast, an unstable protein reaches equilibrium rapidly and its level changes in steps. *Right*: Extrinsic and intrinsic noise predicted for an unstable protein as a function of β. Solid lines are predictions from (G.6) and (G.10), which agree with estimates from 20,000 Monte Carlo simulations. Parameters taken as $\gamma_x = 10hr^{-1}$, geometric burst with $\langle B\rangle = 6$, and $f = 2$. Burst frequency is changed to have a constant mean protein level of 100 molecules for different values of β. 95% confidence intervals are calculated via bootstrapping.

Appendix H

Simulation results

In this section we compare the analytic results obtained in this paper with numerical simulations. We start with results obtained in equation (39). This equation is obtained by assuming that cell cycle times are independent identically distributed random variables. Here we perturb this assumption: suppose cell cycle times $T_i,\ i \in \mathbb{N}$ are related through an AR process as

$$T_i = T_0 + \phi T_{i-1} + \eta_i, \tag{H.1}$$

where η_i is a normally distributed noise with zero mean and variance σ_η^2; moreover T_0 is a constant, and $|\phi| < 1$. For this model the mean and variance of cell cycle time is

$$\langle T_i \rangle = \frac{T_0}{1-\phi},\ i \in \mathbb{N}, \qquad Var(T_i) = \frac{\sigma_\eta^2}{1-\phi^2}. \tag{H.2}$$

Further the cross correlation between two cell cycles which are i cycles apart is ϕ^i. Note that η_i's are independent and identically distributed random variables hence $\langle \eta_i \eta_j \rangle = 0$.

In order to separate noise levels contributed from different sources, we used three models as shown in Figure 2 in the main text. For each model we run 10000 simulations, while model in Figure 2B gives the extrinsic noise, the difference in the noise levels obtained from models in Figure 2B and 2C gives the noise raised from partitioning errors. Nose raised from stochastic protein production can be obtained by subtracting noise levels of the models in Figure 2A and 2C. The comparison between numerical simulations and analytic results based on equation (39) is shown in Figure E. As it is obvious from this

figure our equations match the simulations.

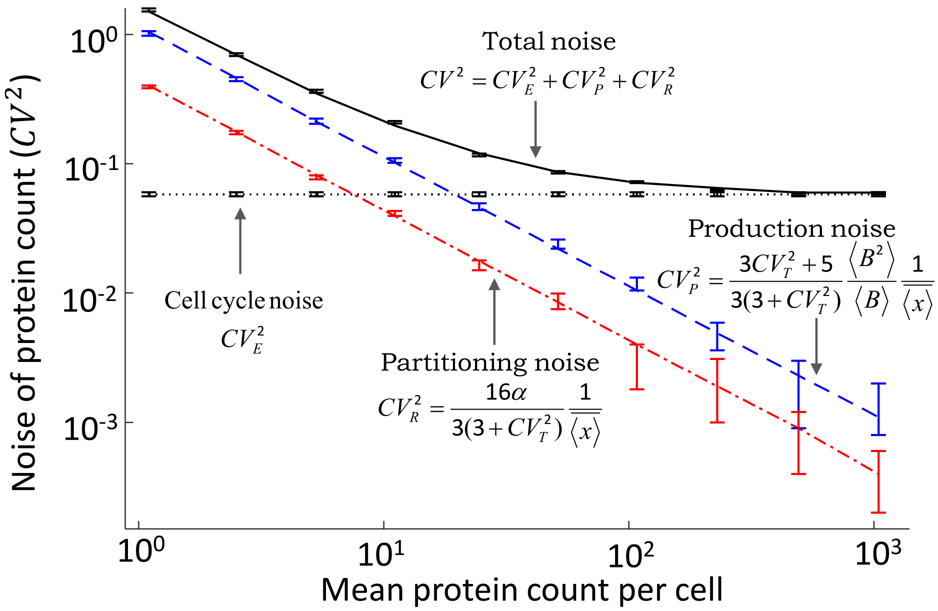

Figure E: **Stochastic simulations show analysis obtained for independent cell cycles can be applied to dependent cell cycle times.** For this plot, B is assumed to be geometrically-distributed with mean $\langle B \rangle = 1.5$. Mean cell cycle time is $20mins$ and $CV_T^2 = 0.05$. We assumed binomial partitioning , i.e., $\alpha = 1$. Error bars obtained via bootstrapping based on 10000 Monte Carlo simulations. For this plot we have assumed a -0.25 correlation between successive cell-cycle times as reported in [5].

Next we use numerical simulations to verify our results obtained for gene duplication. In our analysis we assumed that the time before and after gene duplication are independent. We relax this assumption by assuming that they are correlated, i.e., we draw the first time interval T_1 from a log-normal distribution with mean $\overline{T_1}$ and we assume that the second time interval T_2 is

$$T_2 = \frac{1-\beta}{\beta} T_1 + \eta \tag{H.3}$$

where $\beta = \frac{\langle T_1 \rangle}{\langle T \rangle}$ and η is a normally distributed noise. We fixed the cell cycle time mean $\langle T \rangle = \langle T_1 \rangle + \langle T_2 \rangle = 2\ hours$ [6], and used the models introduced in Figure A to do numerical simulations. We kept correlation coefficient between time intervals before and

after gene duplication around 0.79 [6]. The simulation results for the correlated time intervals is shown in Figure F. The analytic solutions match the numerical results for different gene-duplication times.

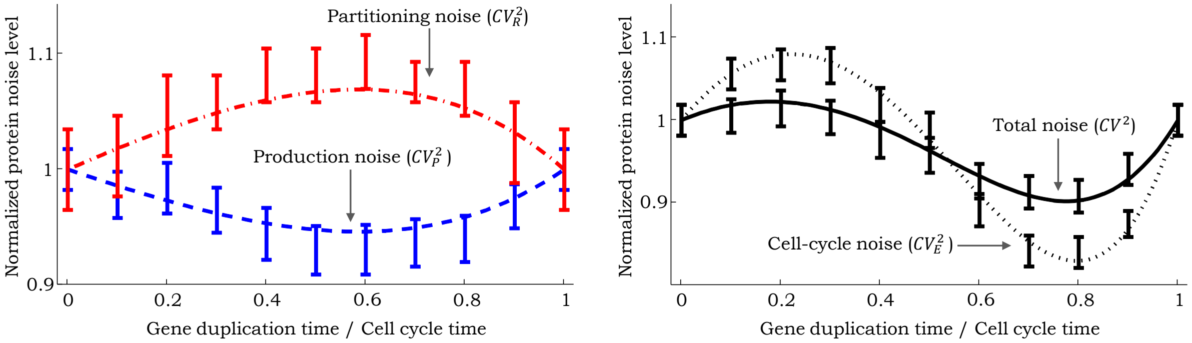

Figure F: **Numerical simulations show results obtained for gene duplication can be applied to dependent T_1 and T_2.** For this plot, B is assumed to be geometrically-distributed with mean $\langle B \rangle = 10$, and mean of protein is set to be 170. Mean cell cycle time is 2 *hours* and $CV_{T_1}^2 = 0.05$. We assumed binomial partitioning , i.e., $\alpha = 1$. The correlation coefficient between time intervals before and after gene duplication is take as 0.79 [6]. Error bars obtained via bootstrapping method based on 10000 Monte Carlo simulations.

Bibliography

1. McQuarrie DA. Stochastic Approach To Chemical Kinetics. Journal of Applied Probability. 1967;4:413–478.

2. Gillespie DT. Approximate Accelerated Stochastic Simulation of Chemically Reacting Systems. Journal of Chemical Physics. 2001;115(4):1716–1733.

3. Hespanha JP, Singh A. Stochastic Models for Chemically reacting Systems Using Polynomial Stochastic Hybrid Systems. International Journal of Robust and Nonlinear Control. 2005;15:669–689.

4. Singh A, Soltani M. Quantifying Intrinsic and Extrinsic Variability in Stochastic Gene Expression Models. PLOS ONE. 2013;8:e84301.

5. Taheri-Araghi S, Bradde S, Sauls JT, Hill NS, Levin PA, Paulsson J, et al. Cell-Size Control and Homeostasis in Bacteria. Current Biology. 2015;25:385–391.

6. Adiciptaningrum A, Osella M, Moolman MC, Cosentino Lagomarsino M, Tans SJ. Stochasticity and homeostasis in the E. coli replication and division cycle. Scientific Reports. 2015;5:18261.
